# Fallopian tube lesions as potential precursors of early ovarian cancer: a comprehensive proteomic analysis

**DOI:** 10.1038/s41419-023-06165-5

**Published:** 2023-09-30

**Authors:** Maxence Wisztorski, Soulaimane Aboulouard, Lucas Roussel, Marie Duhamel, Philippe Saudemont, Tristan Cardon, Fabrice Narducci, Yves-Marie Robin, Anne-Sophie Lemaire, Delphine Bertin, Nawale Hajjaji, Firas Kobeissy, Eric Leblanc, Isabelle Fournier, Michel Salzet

**Affiliations:** 1 Univ.Lille, Inserm, CHU Lille, U-1192 – Laboratoire Protéomique Réponse Inflammatoire Spectrométrie de Masse - PRISM, F-59000 Lille, France; 2 Department of Gynecology Oncology, Oscar Lambret Cancer Center, 59020 Lille, France; 3 Medical Oncology Department, Oscar Lambret Cancer Center, 59020 Lille, France; 4 Department of Neurobiology, Center for Neurotrauma, Multiomics & Biomarkers (CNMB), MorehouseSchool of Medicine, Atlanta, GA 30310 USA; 5 Department of Biochemistry and Molecular Genetics, Faculty of Medicine, American University of Beirut, Beirut, Lebanon; 6 Institut Universitaire de France, 75000 Paris, France

**Keywords:** Predictive markers, Proteomics, Oncogenesis

## Abstract

Ovarian cancer is the leading cause of death from gynecologic cancer worldwide. High-grade serous carcinoma (HGSC) is the most common and deadliest subtype of ovarian cancer. While the origin of ovarian tumors is still debated, it has been suggested that HGSC originates from cells in the fallopian tube epithelium (FTE), specifically the epithelial cells in the region of the tubal-peritoneal junction. Three main lesions, p53 signatures, STILs, and STICs, have been defined based on the immunohistochemistry (IHC) pattern of p53 and Ki67 markers and the architectural alterations of the cells, using the Sectioning and Extensively Examining the Fimbriated End Protocol. In this study, we performed an in-depth proteomic analysis of these pre-neoplastic epithelial lesions guided by mass spectrometry imaging and IHC. We evaluated specific markers related to each preneoplastic lesion. The study identified specific lesion markers, such as CAVIN1, Emilin2, and FBLN5. We also used SpiderMass technology to perform a lipidomic analysis and identified the specific presence of specific lipids signature including dietary Fatty acids precursors in lesions. Our study provides new insights into the molecular mechanisms underlying the progression of ovarian cancer and confirms the fimbria origin of HGSC.

## Facts

1. The better understanding of the biology of pre-neoplastic lesions in the fallopian tube and their role in the development and progression of high-grade serous ovarian cancer.
2. The definition and diagnostic criteria for preneoplastic lesions in the fallopian tube need to be more explicitly outlined to improve consistency across studies and facilitate accurate diagnosis.
3. The development and optimization of on-tissue digestion and microextraction strategies for peptide identification on tissue should continue to be explored to improve spatially resolved proteomics approaches.
4. Future studies should focus on identifying additional biomarkers and developing new analytical techniques to improve the sensitivity and specificity of early detection and diagnosis of high-grade serous ovarian cancer.

## Introduction

Ovarian carcinoma (OC) ranks as the eighth leading cause of cancer in women worldwide in 2020, responsible for approximately 207,000 deaths out of 314,000 cases in 2020. Amongst the different classes, high-grade serous carcinoma (HGSC), is the most represented (> 75%) and within this class, 10% of the patients are mutated for *BRCA1/2* or present with Lynch syndrome [1–3]. While the general population has a 1.2–1.4% risk of developing OC, women with familial BRCA mutations have a significantly higher risk of up to 46%, even at a younger age (between 30 and 35 years old).

Various theories have been proposed regarding the origin of HGSC, some of which suggest the involvement of the epithelial cells in the region of the tubal-peritoneal junction [4–8]. During the last 10 years, special attention has been geared towards pathological examination of operative specimens of early OC and prophylactic bilateral adnexectomy performed in women at risk of OC due to an inherited mutation in *BRCA* genes. Based on Sectioning and Extensively Examining the Fimbriated End Protocol (SEE-FIM) [6], a systematic serial examination of the fallopian tubes is performed, coupled with IHC evaluation of p53 and Ki-67 expression. In this group of patients, an unusual rate of certain cancer or at least cellular abnormalities was observed in the fallopian tube epithelium (FTE), especially at its terminal end, the fimbria [9, 10].

This junction occurs where the peritoneum covers the serosal surface of the fallopian tube and meets the specialized epithelium of the tubal fimbria and is marked by a transition between the Müllerian and the mesothelial cells [11]. Junctional sites between different types of epithelia are known to be cancer hot spots where neoplastic transformations can occur. This is supported by evidence that cells derived from this transition region exhibit a cancer-prone stem cell phenotype [4, 8, 11]. During the neoplastic transformation in the fallopian tube cells, mutations and/or aberrant morphological features can be observed. These neoplastic lesions have been classified into three main groups: p53 signatures, serous tubal intraepithelial lesions (STIL), and serous tubal intraepithelial carcinoma (STIC). These lesions are defined according to the immunohistochemical (IHC) pattern of p53 and Ki67 markers and the architectural alterations of the cells. The p53 signature is identified by a small number of epithelial cells (10–20) with a proliferation activity like the adjacent normal epithelium, but with a p53 staining pattern corresponding to a missense TP53 mutation. STIC (serous tubal intraepithelial carcinoma) involves many cells with architectural and nuclear alterations, TP53 mutations, and high proliferative activity. STILs (serous tubal intraepithelial lesions) are defined by the accumulation of p53 in more than 20 cells, some of which exhibit morphological abnormalities, and a higher Ki-67 proliferating index (10–40%). STIL is characterized by a lower level of abnormalities compared to STIC along with normal proliferative activity [12, 13].

Series of morphological changes concomitant with multi-step accumulation of molecular and genetic alterations on pre-neoplastic lesions of FTE hypothesized that HGSCs derive their origin, not in the ovary, but in the fimbria part of the fallopian tube [14–16]. PAX8, a Mullerian marker is expressed in most HGSC but not calretinin (mesothelial marker) [17, 18]. This view has not been universally accepted, primarily as it conflicts with traditional theories on the origins of OC, and secondarily owing to variation in the detection of tubal lesions in association with HGSC, which may in turn be due to differences in sampling or difficulties in diagnostic interpretation [19, 20]. Recent studies have provided a stepwise progression of FTE to precursor lesions to carcinoma, with the aid of ‑ p53 signature ‑ STIL - STIC - HGSC sequence’s model [15, 19–22].

Our study aims to investigate the molecular mechanisms associated with the spectrum of pre-neoplastic lesions found in the fallopian tube epithelium (FTE). Due to the limited cellularity of these lesions, we performed a spatially resolved proteomics analysis on a discovery cohort consisting of eight patients. Some of the markers identified were validated by immunofluorescence. We also conducted a lipidomic study using SpiderMass technology on a cohort of 27 patients. Finally, we correlated the proteomic and lipidomic markers to better understand the underlying mechanisms. To identify the molecular pathways associated with the different lesions, we have implemented a state-of-the-art spatially resolved proteomics workflow previously validated on different types of cancers [23–25]. However, this work has a novel component by using a pathologist-guided IHC slide of formalin-fixed paraffin-embedded (FFPE) tissue section of patients presented p53 signature lesions, STIL lesions, and STIC lesions which will be compared to non-pathological fallopian tissues and HGSC. Here, we investigate the different pre-neoplastic lesions, their proteome, the mutations detected in proteins, the proteome translated from alternative ORF, so-called the Ghost proteome, and finally construct proteome pathways indicative of underlying mechanisms [26, 27].

We identified several early-stage markers known to be associated in OC with poor (Emilin2, and CAVIN1) or good prognosis (CAVIN2, SPTAN1, FBLN5). Using IHC, we validated the higher expression of CAVIN1/2, SPTAN1, FBLN-5, and Emilin2 in p53, STIL lesions compared to normal FTE and STIC. Finally, we used ambient ionization mass spectrometry with Spidermass technology to perform an analysis of lipidomic profiles on FFPE tissues after dewaxing. The lipidomic data cross-validate the proteomic results, and we identified several discriminative lipids of the p53 signatures, STILs, and STICs, which were related to previously identified pathways of FTE lesions.

## Experimental procedures

### Reagents and chemicals

High-purity chemicals from various suppliers were purchased for the different experiments and used as received without any further modifications. Acetonitrile (ACN) HPLC grade, Ethanol HPLC grade, Methanol LC-MS grade, and Isopropyl alcohol LC-MS were purchased from CARLO ERBA Reagents SAS (Val de Reuil, France). Water UHPLC-MS was supplied by Fisher Scientific (Illkirch, France). Trifluoroacetic acid and Formic Acid (FA) were obtained from Biosolve B.V. (Valkenswaard, the Netherlands). Additionally, alpha-Cyano-4-hydroxycinnamic acid (4-HCCA), Aniline (ANI), and Glycerol were sourced from Sigma Aldrich (Saint-Quentin-Fallavier, France).

### Experimental design, case selection and statistical rationale

Tissue samples were obtained from patients who participated in a multicentric phase II clinical trial of radical fimbriectomy initiated by Oscar Lambret Cancer Centre in Lille, France. Before enrollment, the trial protocol (NCT-01608074) was approved by the national and institutional review boards (IRB) and accepted by the ethics committee (CPP Nord Ouest IV, Dec. 2010) following the French and European legislation.

### Ethics approval and consent to participate

Patients provided written informed consent before participating in the trial. To protect patient privacy, no personal information was used in these experiments, and a random number was assigned to each sample. For more information about the participation criteria and study plan for this clinical trial, please refer to the Clinical Trials website (https://clinicaltrials.gov/ct2/show/NCT01608074). We took necessary measures to ensure that diagnostic lesions were not lost for subsequent clinical follow-up. In our spatially resolved proteomics approach, we used a slide that had already been used in IHC for our analyses. The sample size for this study was limited due to the rarity of the target population, which consists of patients with BRCA mutations undergoing prophylactic adnexectomy, as well as the need for a sufficient sample size to evaluate the feasibility and safety of the surgical procedure. It is also worth noting that not all recruited patients presented with preneoplastic lesions. However, it should be emphasized that there is currently no available information on the prevalence of preneoplastic lesions in the fimbria of patients with *BRCA1 mutations* or family histories who undergo prophylactic adnexectomy. In our study, we performed proteomic analysis using a relatively small number of patients. It is worth noting that increasing the number of samples is not always the optimal solution, as other important factors such as ethical considerations, resource availability, and sustainability need to be considered. Moreover, previous studies have shown that even with a limited number of samples, statistically significant results can still be obtained. Therefore, in our study, we chose to prioritize the quality of the samples to ensure accurate and reliable results for the discovery part and increase the number of patients for the validation part. A prospective cohort of 8 patients was selected to perform proteomics analysis, including 4 patients with p53 signature lesions, 2 patients with STIL lesions, 2 patients with STIC lesions, and 1 patient with HGSC (see Table 1a). Normal tissue was analyzed from the normal part of the tissue section from patients with p53 signature lesions and no other concomitant lesions. Afterward, a retrospective cohort of 27 patients was used (see Table 1b) for Spidermass analysis.

**Table 1:** a: Description of clinical characteristics of patients with a preneoplastic lesion in the fallopian tube fimbria (prospective cohort). b: Description of clinical characteristics of patients with a preneoplastic lesion in the fallopian tube fimbria (validation cohort).

Case ID	Lesions sampled	BRCA status	Age at diagnosis	Location in fimbria	Ovary lesion	Other lesions	Personal history of breast cancer	Other remarks
**a**								
1	Normal, p53 signature	BRCA1	40	Right	No	SCOUT in tube	No	Left ovarian theca cyst
2	Normal, p53 signature	BRCA1	39	Right and left	No	No	Yes	
3	STIL (x3)	unknown	61	Left	No	No	No	Uterus Leiomyoma
4	STIC (x3)	BRCA1	36	Right and left	No	Several p53 signature	No	HGSC in the left tube
5	Normal, p53 signature	Family history	38	Left	No	No	No	
6	STIL, STIC	BRCA1	48	Left	No	Several p53 signature	Yes	
7	Normal, p53 signature	BRCA1	35	Left	No	Several p53 signature	Yes	HGSC in the left fimbria
8	Patient 8 shows an ovarian high-grade serous carcinoma with no evidence of pre-neoplastic lesion in the fallopian tube. The BRCA status is unknown.							
**b**								
F1	STIL	BRCA2	65	Left	No	No		
F2	Normal, p53 signature	BRCA1	38	Right	No	No	Yes	
F3	STIC	BRCA1	37	Right	No	STIL to the left fimbria	Yes	HGSC in the left tube
F4	Normal, p53 signature	unknown	50	Left	No	No	No	Follicular cyst in the ovary
F5	Normal, p53 signature	BRCA2	50	Both sides	No	No	Lesion in the breast	
F6	STIC	BRCA1	41	Right	No	No	No	cyst in the left ovary
F7	Normal, p53 signature	No	45	Right	No	HGSC in the left tube		Family breast and ovarian cancer
F8	Normal, p53 signature	BRCA2	67	Left	No	No	No	
F9	Normal, p53 signature	BRCA1	70	Both sides	No	No	No	
F10	STIL, STIC	BRCA1	62	Both sides	HGSC in both ovaries	No	No	
F11	STIC	BRCA1	44	Right	No	No	Yes	
F12	Normal, p53 signature	No	68	Right	No	No	No	
F13	Normal, p53 signature	unknown	62	Right	Brenner tumor in right ovary	No	Yes	
F14	STIC	BRCA1	44	Right	No	HGSC in the right chorion	Yes	
F15	Normal, p53 signature	BRCA2	46	Left	No	No	No	
F16	Normal, p53 signature	BRCA2	50	Left	No	No	Yes	
F17	STIC	BRCA1	44	Right	No	No	No	
F18	STIL	BRCA2	49	Left	No	No	No	
F19	Normal, p53 signature	BRCA2	43	Right	No	No	No	
F20	Normal, p53 signature	unknown	51	Right	No	No	Yes	Family breast and ovary cancers
F21	Normal, p53 signature	BRCA2	41	Right	No	No	No	
F22	STIL	BRCA1	58	Right	No	No	Yes	
F23	Normal, p53 signature	BRCA1	60	Left	No	HGSC in the right tube	Yes	
F24	STIC	BRCA1	42	Right	No	No	Yes	
F25	STIC, STIL	BRCA1	68	Both sides	No	No	No	
F26	STIL	BRCA1	66	Left	No	No	Yes	
F27	STIL, STIC	unknown	35	Both sides	No	HGSC in the left tube	Yes	

### Sample processing

Our study analyzed FFPE tissue obtained from prophylactic adnexectomies of women with *BRCA1* mutations or family history. Our study was performed on patients presenting with pre-neoplastic lesions without any concomitant ovarian lesions. All samples were examined following SEE-FIM (Sectioning and Extensively Examining the FIMbria) protocol [28]. On these samples, tissues sections of 7 µm thickness were cut using a microtome and were deposited on a glass slide, HPS (Haematoxylin Phloxine Saffron) and immunostaining against P53 (Ventana,790–4286) and Ki-67 (Ventana, 800–2912) were performed and examined by a pathologist to find preneoplastic lesions (p53/STIL/STIC/HGSC) [13].

### MALDI-mass spectrometry analysis

To ensure reproducibility and transparency, we have provided a detailed description of the sample preparation protocol for MALDI MSI and the data analysis methodology in the Supporting Information.

### On-tissue spatially resolved proteomics guided by p53 and Ki67 IHC

**Fig. 1:**
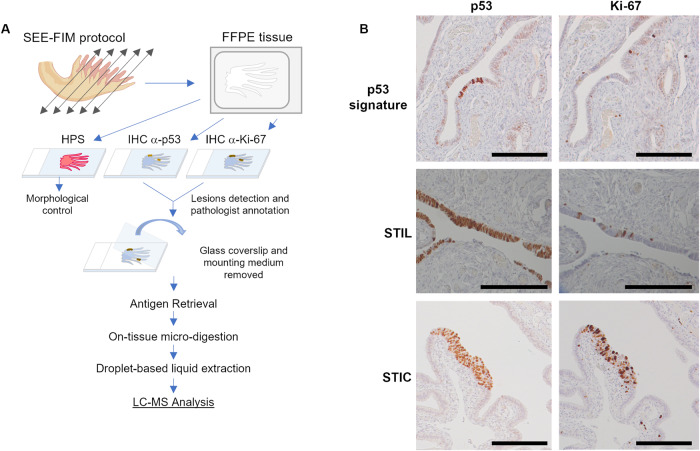
Workflow for spatially resolved proteomic using IHC tissue section. **A** Protocol based on tissue from SEE-FIM protocol. After IHC against p53 and Ki-67, the coverslip glass and the mounting medium are removed to access the tissue section. Digestion of the lesion is performed directly on the tissue section and a droplet-based liquid extraction is performed to recover the peptides before MS-based proteomics analysis. **B** Pre-neoplastic lesions found in the fallopian tube are defined by p53 positivity and Ki-67 index. For p53 signature: accumulation of p53 in at least 12 cells without morphological abnormalities and low Ki-67 index; STIL: same accumulation of p53 in more than 20 cells with some morphological abnormalities and a higher Ki-67 proliferating index (10–40%); STIC: high p53 and Ki-67 index and cells atypical morphology (carcinoma-like).

The complete workflow for sample preparation is illustrated in Fig. 1A.

### Sample preparation

To ensure adequate statistical power and account for potential biological variability, we collected multiple samples from the same patients but on different lesions, resulting in four sampling regions per lesion (Table 1) for proteomics. This approach increases the number of replicates, thus improving the reliability of the statistical analysis. In addition, it allows for a more comprehensive assessment of the heterogeneity within and between different lesions. The same slides as the one used and annotated by the pathologist were unmounted; resin was removed by soaking them overnight in xylene and rinsing them with xylene and ethanol baths. The tissues were rehydrated using 5’ each successive bath of decreasing ethanol degree (2 × 95°, 1 × 30°) and two baths of 10 mM NH4HCO3 buffer. Then, an antigen retrieval step was performed to increase trypsin access to biomolecules. For this, the slides were dipped in 90 °C pH9 20 mM Tris for 30 min, rinsed twice with NH4HCO3, and dried under vacuum at room temperature.

### In situ trypsin digestion

On localized pre-neoplastic lesions, tryptic digestion was performed using a Chemical Inkjet Printer (CHIP-1000, Shimadzu, Kyoto, Japan). The region was carefully selected to ensure that the analysis was restricted to the epithelial cells marked by IHC, thus reducing the potential for contamination from other cell types. The trypsin solution (Sequencing grade modified (Promega), 40 µg/mL, 50 mM NH4HCO3 buffer) was deposited on a region defined to 600 × 600 µm² during 2 h. During this time, the trypsin was changed every half-hour. With 350 cycles and 450 pL per spot, a total of 6.3 µg of trypsin was deposited. To stop digestion, 0.1% TFA was spotted during 25 cycles.

### Liquid extraction

After micro-digestion, the spot content was gathered by liquid microjunction using the TriVersa Nanomate (Advion Biosciences Inc., Ithaca, NY, USA) using Liquid Extraction and Surface Analysis (LESA) settings. With 3 different solvents composed of 0.1% TFA, ACN/0.1% TFA (8:2, v/v), and MeOH/0.1% TFA (7:3, v/v). A complete LESA sequence runs 2 cycles for each mixture composed of an aspiration (2 µL), a mixing onto the tissue, and a dispensing into low-binding tubes. For each interesting spot, 2 sequences are pooled into the same vial.

### MS-based proteomic

A complete description of NanoLC-ESI-MS/MS is provided in supporting information. We implemented a randomization process to minimize the impact of batch effects in our analysis.

### Proteins identification

Raw MS/MS spectra were processed using MaxQuant (version 1.5.6.5, [29]). Peptide identifications were obtained according to a target decoy search against the Homo sapiens Uniprot database (version of 2017_02, 20,172 entries) and a database containing 262 commonly detected contaminants. The human UniProt database was used as the forward database and a reverse one for the decoy search was automatically generated in MaxQuant Mass tolerances were set to 10 ppm and 20 ppm respectively for parent and fragments measurement. Enzyme specificity was set to “trypsin” with a maximum of 2 missed cleavages allowed. Methionine oxidation and acetylation of protein N-terminal were set as variable modifications. FDR < 1% was set for peptides and proteins identification. Two peptides with one unique were necessary to assess protein identification. For label-free quantification, the MaxLFQ algorithm was used. The option “Match Between Runs” was enabled to maximize the number of quantification events across samples. This option allowed the quantification of high-resolution MS1 features not identified in every single measurement. Data generated by MaxQuant were analyzed using Perseus (version 1.6.2.3, [30]). LFQ values were used and proteins were removed if found in the category only identified by site modifications, in the decoy reverse database, or identified in the contaminant database.

### Proteomics statistical analyses

We removed proteins that were not presented in at least three of four replicates of each lesion. We took the average expression per group to perform a comparison and visualized using a Venn diagram. Individual LFQ values were used to perform a multi-scatter plot and calculate a Pearson Correlation between samples. Missing values were imputed based on the normal distribution (width = 0.3, down-shift = 1.8). Principal component analysis (PCA) was done to compare the protein content of each sample. An ANOVA Multi-sample test was performed and consolidated by a Permutation-based FDR (FDR < 0.05, 250 randomizations). A specific comparison between normal and p53 signature samples was performed using a student’s T-test. The differentially expressed proteins with significant differences were selected (values were z-score). The proteomic samples were then clustered according to a Euclidean average as a distance measure for column and row clustering. Up-and-down-regulated proteins in the different groups were utilized to perform an annotation analysis of gene ontology terms by using Funrich (v3.1.3, [31]). A hypergeometric test was performed against all annotated gene/protein lists by comparing the multiple datasets. Enrichment for biological process, transcription factor, and cellular component were presented as a bar chart. PANTHER Classification System (v14.1) was also used. PANTHER Overrepresentation test (downloaded on 20190701) was performed using each list of up-or down-regulated proteins as “analyzed list” and Homo sapiens as “reference list”. Fisher’s Exact test with false discovery rate (FDR) correction was also used. Subnetwork Enrichment Analysis (SNEA) from Elsevier’s PathwayStudio version 10.0 (Elsevier). was used to extract statistically significant altered biological and functional pathways in the different clusters of proteins. The immunohistochemical (IHC) data of different proteins of interest were investigated from the Human Protein Atlas (HPA) database (http://www.proteinatlas.org) [32]. Evaluation of the prognostic effects of these proteins on overall survival (OS) was also extracted from HPA. Comparisons are performed with the 20 genes of the highest significance associated with unfavorable prognosis for ovarian cancer, cervix cancer, endometrial cancer, and breast cancer.

### Mutation identification

The raw MS data were also processed to search for potential mutated peptides using the XMAn database [33]. This database contains information on mutations observed in cancers as well as other diseases. Proteome Discoverer 2.1 (PD 2.1) was used to query the data using MS Amanda as a search node against the XMAn database, the human Uniprot database, and a database containing potential contaminants. Peptides identified only in the XMAn database at a high level of confidence were selected, and the MSMS spectra were manually inspected to confirm the presence of the mutation.

### Alternative proteins (AltProts) identification

RAW data obtained by nanoLC-MS/MS analysis were analyzed with Proteome Discoverer V2.3 (Thermo Scientific) using the Label-Free Quantification node, the protein database is downloaded from Openprot (https://openprot.org/, [34]). This database included RefProt, novel isoforms, and AltProts predicted from both Ensembl and RefSeq annotations (Ensembl: GRCh38.83, RefSeq: GRCh38p7) for a total of 658,263 entries. The following processing and consensus parameters are used: Trypsin as an enzyme, 2 missed cleavages, methionine oxidation as variable modification, precursor mass tolerance: 10 ppm, and fragment mass tolerance: 0.1 Da. The validation was performed using Percolator with a protein-strict FDR set to 0.001. A consensus workflow was then applied for the statistical arrangement, using the high-confidence protein identification. Results are filtered to keep the master protein and high-confidence protein FDR. Then results are extracted in a table, to use the LFQ values in PERSEUS, where an ANOVA statistical test is performed, and the results are represented by a diagram as the heatmap described above.

### Confirmatory immunohistochemistry analyses

Early prognosis validation was performed using antibodies directed against CAVIN2 (Rockland, 600-401-J31), Emilin2 (Thermo Scientific, pa584657), CAVIN1 (Abnova, H00284119-M02), SPTAN1 (OriGene, TA812019), and FBLN5 (Invitrogen, MA5-42544). After dewaxing and antigen retrieval with citrate buffer, the tissues were incubated with a primary antibody at 4 °C overnight, followed by the application of a secondary antibody (Alexa fluor conjugated antibody, 1/200 dilution) for 1 h at RT. We used the following primary antibodies and dilutions: Cavin-1 (1/300), Cavin-2 (1/100), SPTAN1 (1/2000), Emilin-2 (1/500), Fibulin-5 (1/500). All slides were imaged on the Zeiss LSM700 confocal microscope. Three pictures were taken for each tumor section.

### Lipidomic analyses through Spidermass technology

A complete description of Spidermass is provided in the Supporting information.

### SpiderMass analysis of FFPE tissues

Tissues were sectioned at 7 µm thickness and deposited onto a polylysine glass slide. Then, the FFPE tissue sections were submitted to dewaxing two times in xylene for 5 min and were manually sprayed with a glycerol/isopropyl alcohol (IPA) (8:2, v/v) solution in 2 successive passes using a manual sprayer (Agilent). The syringe pump (74 900 series Cole Parmer Instrument Company) was set to a 700 µL/min flow rate. The 2 successive passes were equal to 5 µL deposited on 1 cm2 and took ∼10 s. The samples were analyzed within 10 min after the glycerol deposition52.

### Classification model from SpiderMass data

For data analysis, all raw data files produced with the SpiderMass instrument were imported into the Abstract Model Builder (AMX v.0.9.2092.0) software. After importation, spectra were first pre-processed. The pre-processing steps include background subtraction, total ion count normalization, lock mass correction, and re-binning to a 0.1 Da window. All processed MS spectra obtained from the 27 histologically validated samples were then used to build a principal component analysis and linear discriminant analysis (PCA-LDA) classification model51. The first step consisted of PCA to reduce data multidimensionality by generating features that explain most of the variance observed. These features were then subjected to supervised analysis using LDA by setting the classes that the model will be based upon. LDA attempts to classify the sample spectra and assess the model by cross-validation. Cross-validation was carried out by either using the “20% out” or the “leave one patient out” methods. For the first method, 20% of MS spectra are randomly taken from the total spectra, and the model is reconstructed from the remaining 80%. The remaining 20% of spectra are used to interrogate the reconstructed model. The permutation is automatically reiterated for 5 cycles before reporting the cross-validation results. For the second method, the spectra are grouped by patients and left out one by one; at each step, the model without the patient is interrogated against this model.

## Results

### In situ spatially resolved proteomics of FTE lesions

The primary goal of the clinical trial investigated the effectiveness of bilateral laparoscopic radical fimbriectomy in eliminating the potential source of dysplastic cells in the fallopian tubes while maintaining ovarian hormonal function [35]. A second part aimed to identify a potential proteomic timeline associated with ovarian cancer progression and development. Given the scarcity of the preneoplastic lesion, only a limited number of patients were recruited for the clinical trial. To optimize the use of tissue samples, we decided to reuse the previously immunostained FFPE tissue sections using highly refined mass spectrometry-based methods (Fig. 1A). A cohort of 8 patients presenting *BRCA 1* mutations or hereditary susceptibility, was selected for the spatially resolved proteomic experiments (Table 1a). These experiments aimed to target the preneoplastic lesions, which are only present in very few cells already under transformation in small areas of the tissues. To confirm the presence of these lesions, a double IHC against p53 and Ki-67 markers is needed (Fig. 1B). To enable spatially resolved proteomics, we developed a new protocol that uses the same IHC slide as the pathologist, which had previously served for the detection of the lesions (Fig. 1A). Targeting these specific lesions allows for precise localization of the affected cells and decreases the presence of proteins for normal tissue in the samples.

To investigate the possibility of identifying specific protein signals from different lesions, we conducted MALDI mass spectrometry imaging (MALDI-MSI) experiments. Our findings, as shown in Supplementary Fig. S1, revealed the presence of specific ions corresponding to proteins that were specific to the STIC lesion, such as m/z 3019.19 and m/z 2639.76, which were only found in the tumor area. To highlight the main molecular regions of the tissue, we performed a spatial segmentation, which revealed a statistically significant difference between the tumor region and the non-tumoral region of the tissue Specifically, we identified a specific cluster (light blue) in the tumor region (Supplementary Fig. S1B). Moreover, our segmentation of MALDI-MSI data from two tissues, one with no lesion and one with a p53 signature, also showed the presence of a specific cluster (orange) with a different molecular profile within the area where the lesion was identified by IHC (Supplementary Fig. S1C). These findings suggest that specific protein signals can be identified from different lesions, providing a foundation for spatial proteomics analysis.

**Fig. 2:**
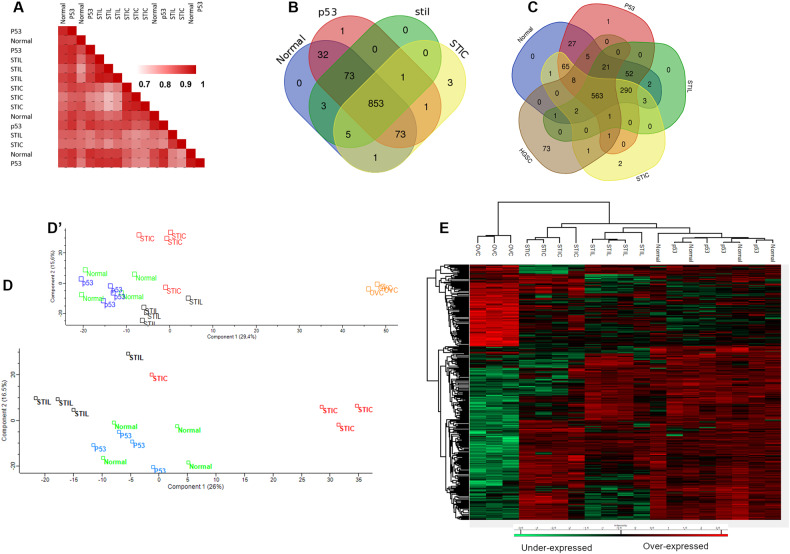
Comparison of the different lesions based on their proteomic signature. **A** Correlation analysis based on the whole proteome. Ven diagrams showing the distribution of identified proteins among (**B**) normal tissue and the different lesions and (**C**) with the addition of HGSC data. Principal component analysis using (**D**) normal tissue and the pre-neoplastic lesions and (**D’**) with addition to HGSC. **E** Hierarchical clustering of the most variable proteins (ANOVA with permutation-based FDR < 0.05) including HGSC data.

Then, the IHC slides stained for p53 and Ki67 were directly used to perform the spatial shot gun proteomic experiments from all the patients and tissue sections. After marking the area and unmounting the coverslip, the tissues were submitted once more to AR followed by a micro-digestion with trypsin and a micro-extraction (see picture, Fig. 1A and Supplementary Fig. S1D). Our workflow identified 10,375 unique peptide sequences corresponding to 1,617 distinct protein groups across pre-neoplastic lesions and non-pathological tissues. Label-free quantification (LFQ) of proteins resulted in a total of 1571 proteins quantified (Fig. 1C). After filtering the proteins according to a minimum number of values (2/3 of valid values) in at least one group of the four defined groups (normal, p53 signature, STIL, and STIC), we retained 1,046 proteins for further analysis. The number of identified proteins is consistent with our previous experience on FFPE tissue, considering that the tissues were stored over many years after fixation [36]. Notably, the spatial proteomic analysis was unaffected by the removal of the coverslip glass and mounting medium from the IHC sections evaluated by the pathologist. To validate these findings, we conducted a correlation analysis of all quantified proteins. The protein patterns of samples from the same group compared with inter-group variation were similar (mean Pearson correlation 0.92). The main differences were observed between STIC and STIL lesions (mean Pearson correlation 0.84) (Fig. 2A). Although our sample size was limited, these findings are consistent with the inter-patient variability typically observed, indicating that our protocol did not significantly alter the proteomic content of the tissue sections. This allows us to confidently perform a comparative analysis to obtain a specific proteomic signature of the different lesions.

### Comparison of the proteomic profiles of p53 signature, STIL, STIC, and normal fimbria

The protein content of each group was compared by averaging the replicates and using a Venn diagram representation. It was observed that 853 common proteins (81.5% of overlap) were presented between normal tissues and pre-neoplastic lesions (Fig. 2B, Supplementary Data 1). Three proteins were found to be exclusive to the STIC lesions and one protein was identified only in the p53 signature lesion. Among these, RNA-binding protein 10 (RBM10) was found in the p53 signature lesions and not in the normal tissue or other lesions. RBM10 is an RNA-binding protein located in the nucleus that plays a crucial role in RNA splicing and is implicated in several human diseases, including cancer. Three proteins were found exclusively in the STIC lesions: Glucosamine (N-acetyl)-6-sulfatase (GNS), Upstream binding transcription factor, RNA polymerase I (UBTF), and ATPase family, AAA domain containing 3 (ATAD3A/B). According to data from the Human Protein Atlas (HPA), RBM10, which was found exclusively in the p53 signature lesion, is overexpressed in cervical and endometrial cancers and has a favorable prognostic value. On the other hand, the elevated expression of GNS and ATAD3A/B is associated with poor survival rates in liver cancer, while high expression of GNS in glioma and ovarian cancer is a marker of poor prognosis. However, the expression of UBTF appears to have no impact on survival. Regarding the HPA data on the identified proteins, it should be noted that while these proteins are indicated as highly expressed in the fallopian tube, this expression is primarily detected in the glandular cells. These cells are found in higher proportions near the uterus, whereas cilia cells are more represented in the fimbria part. Therefore, the expression levels of these proteins may differ depending on the specific region of the fallopian tube being analyzed. The inclusion of HGSC data in the analysis (Fig. 2C, Supplementary data 2) revealed that UBTF was expressed both in STIC lesions and HGSC. Fibrillarin (FBL) is found only in pre-cancerous lesions and not in normal tissue. This protein is also observed in HGSC (Fig. 2C, Supplementary Data 2).

There were a limited number of proteins exclusively identified in each group, indicating that the variation is primarily in protein abundance rather than specific protein composition. To better understand the modulation registered across the different lesions, we performed principal component analysis (PCA) (Fig. 2D). Components 1 and 2 represent 42.5% of the total data variation. Samples from normal tissue and p53 signature are difficult to discriminate. However, samples from STIL and STIC lesions were different from normal and p53 signature samples in component 2. Interestingly, STIL lesions were closer to normal and p53signature lesions in component 1, compared to STIC samples. We further confirmed that the proteome of ovarian HGSC tumors is highly different compared to STIL, STIC, p53 signature, and normal tissues (Fig. 2D’). Then, we performed hierarchical clustering to analyze the significant differences in protein expression between the five groups (Fig. 2E). The results showed that HGSC tumors were separated from the other tissues by one main branch. The second branch is further divided into two sub-branches with one separating STIC lesions from p53 signature/Normal/STIL lesions, and the other separating STIL lesions from p53 signature/Normal tissues (Fig. 2E). As previously observed, the last group (p53/Normal) was challenging to differentiate. Nevertheless, the molecular differences in cell composition between the ovary and the fallopian tube did not allow us to draw a direct conclusion on the relationship between HGSC and the pre-neoplastic lesions of the fallopian tube. However, we demonstrated that the proteomic profile of the STIC lesions was closer to HGSC compared to the other lesions, and each lesion had a specific proteomic signature.

### Descriptive proteomics analysis of preneoplastic lesions

**Fig. 3:**
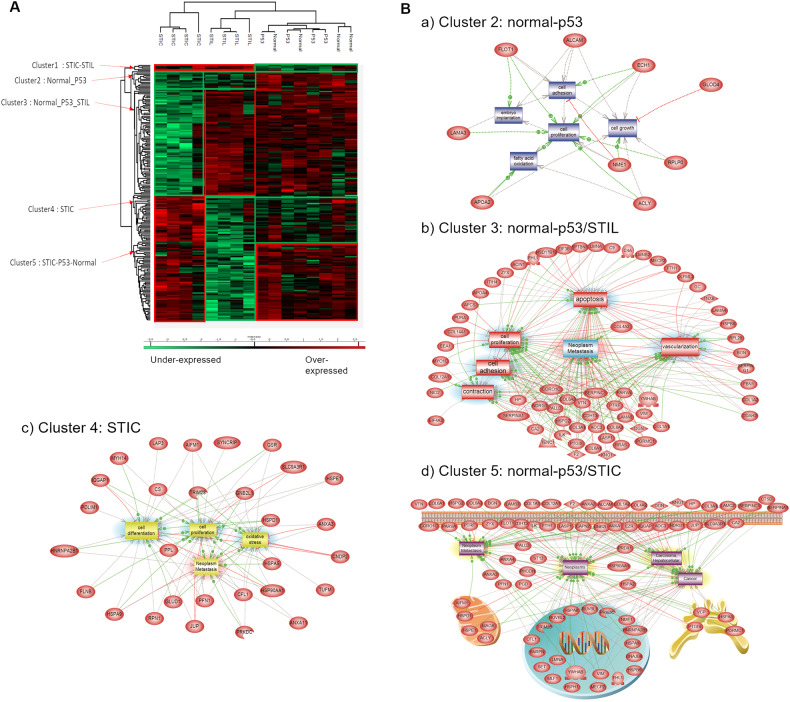
Proteomic analysis of the pre-neoplastic lesions. **A** Hierarchical clustering of the most variable proteins between normal tissue, p53 signature, STIL and STIC (*n* = 4 for each category, ANOVA with permutation-based FDR < 0.05); **B** Subnetwork Enrichment Analysis was done to highlight altered biological and functional pathways in the different clusters of proteins.

**Fig. 4:**
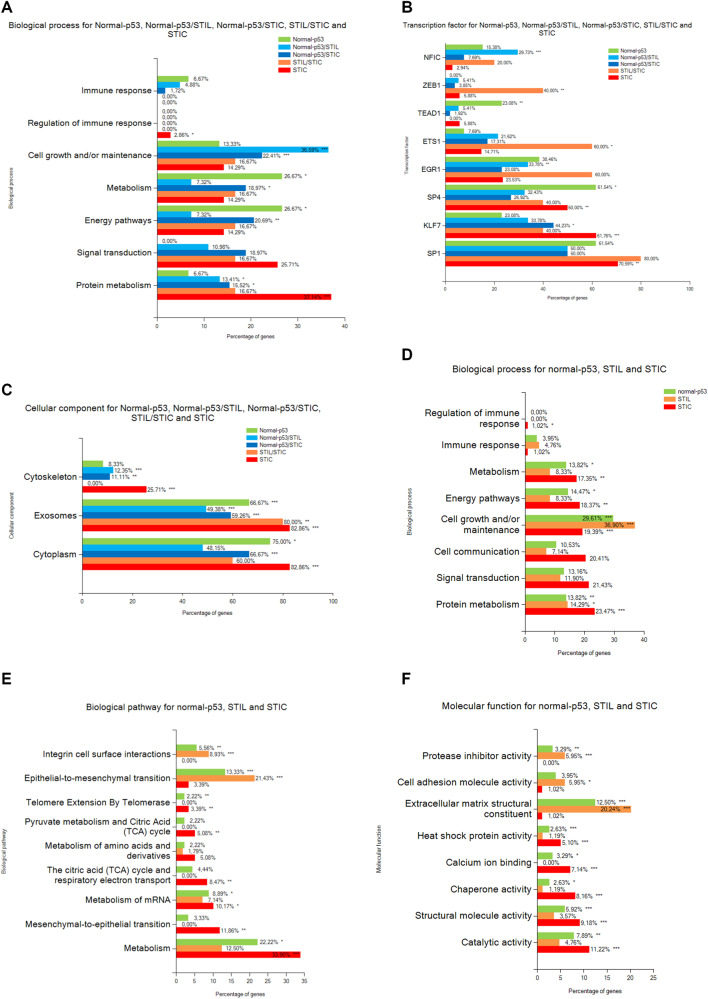
Annotation analysis of gene ontology terms. **A** Biological process, **B** Transcription factor and **C** Cellular component for the clusters of proteins. **D** Biological process, **E** Biological pathway and **F** Molecular function of the proteins overexpressed in each lesion. (hypergeometric test against all annotated gene/protein list of Funrich database, the *p*-value is represented by stars: ***p < 0.001, **p < 0.01, *p < 0.05 no star for *p* > 0.05).

For a deeper analysis of the pre-neoplastic lesions of the FTE, ANOVA testing was performed without the HGSC data. A total of 197 proteins were differentially expressed (significantly different) within the four groups (Supplementary Data 3). A hierarchical clustering based on the expression of these proteins resulted in two main clusters (Fig. 3A, Supplementary Data 3). As observed previously, normal and p53 signatures were not resolved. This group as well as STIL lesions clustered together (Fig. 3A). The proteomic profile of STIC lesions significantly differed from the two other groups. The heatmap displays five main clusters of proteins. Proteins that were enriched in STIL and STIC regions were represented in **cluster 1**. Those that were more abundant in both normal tissue and p53 signature lesions were represented in **cluster 2**. Proteins overexpressed in normal, p53 signature lesion and STIL but not in the STIC were represented in **cluster 3**. Proteins that were overexpressed in the STIC lesions were represented in **cluster 4** and those specific to normal tissue, p53 signature lesion, and STIC were represented in **cluster 5**. **Cluster 1** (STIL/STIC) was composed of only 5 proteins i.e., MARCKS-related protein (MARCKSL1), Myosin-10 (MYH10), Protein SET (SET), Double-stranded RNA-binding staufen homolog-1 (STAU1), and D-3-phosphoglycerate dehydrogenase (PHGDH). The Protein SET participates in numerous cellular functions including DNA repair, transcription, cell survival, and proliferation. This protein is also involved in many cancer processes such as metastasis, the development of therapeutic drug resistance, and play a key role in tumorigenesis [37]. MARCKSL1 is known to promote the progression of lung adenocarcinoma by regulating epithelial-to-mesenchymal transition (EMT) [38]. STAU1 has been previously identified in colorectal cancer [39], whereas PHGDH protein has been identified in ovarian cancer [40]. In **cluster 2** (normal-p53), 15 proteins showed an overexpression for normal and p53 signature lesions. Among these proteins, CD166 antigen (ALCAM/CD166) is shown to be a potential cancer stem cell marker [41]. It has been found as a poor prognostic marker in pancreatic cancer [42]. This protein is known to contribute to local invasion and tumor progression by acting on the detachment of tumor cells [43]. Moreover, TNPO1, ACLY, NME1, FLOT1, and KLC4 are proteins already known to be involved in epithelial ovarian cancer [44, 45]. Gene Ontology (GO) analyses confirmed that these proteins are involved in cell proliferation, adhesion, and growth (Fig. 3Ba). **Cluster 3** (normal-p53/STIL) contains 80 proteins. Proteins involved in complement and coagulation cascades are present in this cluster (SERPIN A1, SERPIN C1, SERPIN G1, C4a, C9, Vitamin D-binding protein (GC), pro-thrombin (F2), Vitronectin (VTN), kininogen (KNG1)) and 10 members of the collagen family. The PGRMC1 (Membrane-associated progesterone receptor component 1) is known to be involved in ovarian cancer [46] as well as PTGIS (Prostacyclin synthase) and the Protein NDRG1 known to modulate genes involved in ovarian cancer metastasis [47, 48]. GO analysis confirmed that these proteins are involved in neoplasia/metastasis, angiogenesis, and apoptosis (Fig. 3Bb). **Cluster 4** (STIC only) contained 36 proteins involved in cell migration such as CFL1 (Cofilin-1), PFN1 (profilin-1), PPL (periplakin), FLNB (filamin B), PDLM1 (PDZ and LIM domain protein 1), IQGAP1 (Ras GTPase-activating-like protein), MYH14 (Myosin-14) and in metabolism (CS (Citrate synthase), DECR1 (2,4-dienoyl-CoA reductase), GSR (Glutathione reductase), LAP3 (Cytosol aminopeptidase), GLUD1 (Glutamate dehydrogenase 1). Proteins of this cluster were involved in cell differentiation, proliferation, and in neoplasia/metastasis (Fig. 3Bc). The last cluster (normal-p53/STIC) was composed of 61 proteins. Several proteins involved in exosomes have been identified (Annexins A1, A2, A4, A13, EZR (ezrin), HSPA8 (Heat shock 71 kDa protein 1 A)). Other proteins are involved in stress such as PRDX1 (Peroxiredoxin-1), HSP8, HSPA1 (Heat shock 70 kDa protein 1), HSPA2 (Heat shock 70 kDa protein 2), ST13 (Hsc70-interacting protein), DNAJB6 (DnaJ homolog subfamily B member 6), VCP (Transitional endoplasmic reticulum ATPase), TUBB4B (Tubulin beta-4B chain). This cluster showed an overrepresentation of proteins involved in neoplasia and cancer transitions (Fig. 3Bd). Functional enrichment analysis points out differences between clusters. Different biological processes have been identified i.e. immune response, regulation of immune response, cell growth and/or maintenance, metabolism, energy pathways, signal transduction and protein metabolism (Fig. 4A). Enriched transcription factors (TFs) that regulate the overexpressed proteins in the different clusters were also obtained (Fig. 4B). The cluster containing proteins overexpressed in normal-p53 signature showed a high abundance of proteins involved in metabolism and energy pathways as well as an enrichment of SP1, SP4 and TEAD1 transcription factors that targeted the most genes of this cluster. For the cluster normal-p53/STIL, proteins were mostly involved in cell growth and/or maintenance and protein metabolism. Enrichment of the TFs NFIC and EGR1 was observed. Concerning Normal-p53/STIC, identified proteins were implicated in cell growth and /or maintenance, metabolism, energy pathways and protein metabolism, while KLF7 protein was identified as a TF. For STIL/STIC cluster, enrichment of the TFs ZEB1 and ETS1 was observed. STIC cluster presented a strong enrichment of proteins involved in protein metabolism and regulation of immune response and SP1, SP4 and KLF7 as TFs. We also observed a reduction of the proteins involved in immune response in STIC lesions. Cellular components analysis reflected that all proteins of the 5 clusters were in the cytoplasm or in exosomes (Fig. 4C) but with some differences. In fact, in STIL-STIC and STIC, we observed a strong enrichment of exosomes, whereas p53 signature and STIC involved cytoplasmic proteins. We also observed a high enrichment of cytoskeletal proteins in STIC lesions. Altogether, these analyses showed molecular transitions occurring between Normal-p53 signature, p53 to STIL and STIL to STIC with a change in proteins involved in cell growth and/or maintenance and the different metabolism processes. To confirm these observations, further analysis were carried out, this time grouping together all proteins overexpressed in each lesion, i.e., normal-p53 signature, STIL, and STIC (Fig. 4D, E, F and Supplementary Data 3). In STIL, an enrichment of proteins involved in cell growth and/or maintenance (Fig. 4D), in integrin cell surface interactions (Fig. 4E), in extracellular matrix (ECM) structural constituent and cell adhesion molecule activity (Fig. 4F) were observed. the ones in various metabolism and energy pathways (Fig. 4D, E) and catalytic activity (Fig. 4F) were lower than for the other cellular transition. We also investigated whether the proteins involved in the Warburg effect [49] were activated to compensate for the overall decrease in the classical metabolism processes observed (Supplementary Fig. S2). These proteins were not altered overall and a trend toward decreasing suggest that no Warburg effect seemed to occur. In STIC, proteins involved in metabolism, energy pathways, cell communication, signal transduction (Fig. 4D, E), calcium ion binding, catalytic and structural molecule activities (Fig. 4F) were higher than in the other transition lesion. In the same time, immune response (Fig. 4D), integrin interactions (Fig. 4E), cell adhesion molecule activity and ECM structural constituent (Fig. 4F) were highly repressed. A statistical overrepresentation test using PANTHER classification system analyses (Supplementary Data 4) confirmed such transition.

**Fig. 5:**
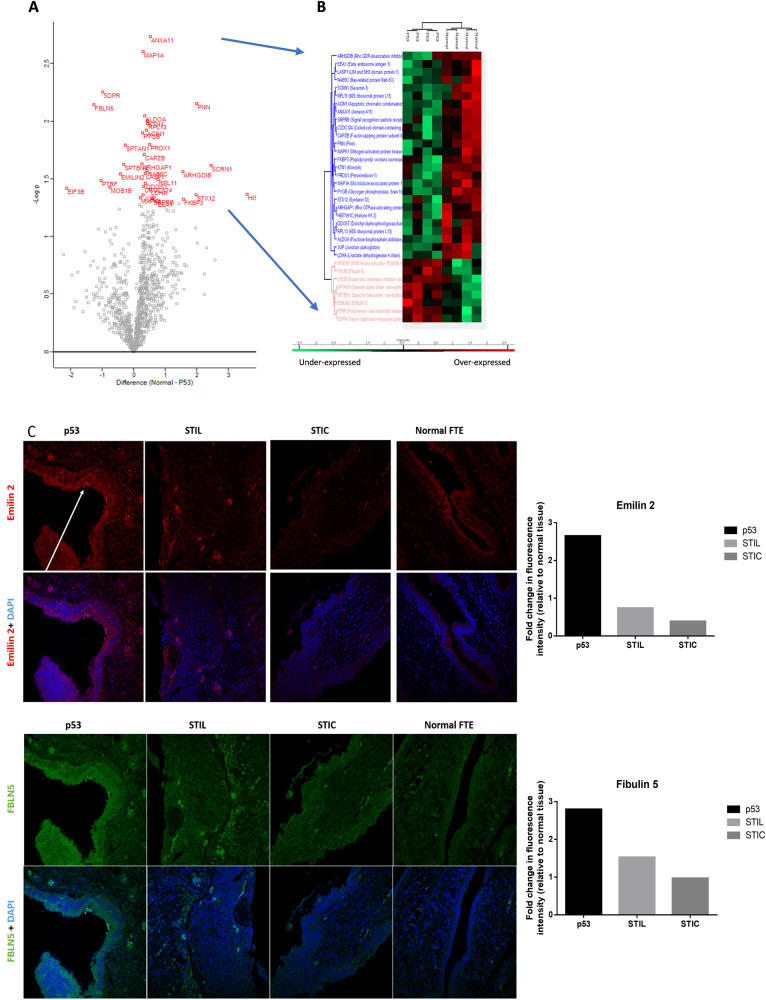
Comparison between p53 signature and normal tissue. **A** Visualization of a t-test in form of a volcano plot comparing normal to p53 lesion (proteins with *p* < 0.05 in red.) **B** Hierarchical clustering of the significant variable proteins between normal tissue and p53 signature (t-test with *p* < 0.05), **C** IHC of p53, STIL and STIC lesions with anti-FBLN5 and anti-Emilin2 antibodies, the arrow shows the p53 lesion on the tissue. The graphs represent the quantification of the fluorescence intensity of each marker in the corresponding tissue relative to the fluorescence intensity in the normal FTE.

Regarding the p53 signature and normal tissue, which could not be distinguished using group analysis, but using a Student’s *T* test, 8 proteins were found to be highly discriminant between Normal and p53 lesions with a specific over-expression in p53 signature (Fig. 5A, B, Supplementary Data 5). Table 2 presents the immunoreactivity in normal FTE and the prognostic effect on overall survival (OS) in OC for these 8 proteins extracted from the HPA. PTRF, also named CAVIN1, is an unfavorable prognosis marker for ovarian, urothelial, and colorectal cancers, whereas SDPR (known as CAVIN2) is associated with a favorable prognosis in renal cancer and a poor prognosis in stomach cancer. The IHC data validation indicated that these proteins are not detected in normal FTE. It also showed a moderate positivity in OC for CAVIN1 and a strong positivity in a rare case of endometrioid carcinoma of the ovary for CAVIN2. SPTAN1 and FBLN5 are favorable prognostic markers in renal cancer but unfavorable in OC (considering a *p* < 0.05). Emilin2 is already known as a poor prognostic marker for liver cancer and a poor prognostic marker for renal and head-neck cancers. By IHC, we validated a higher expression of CAVIN1 in p53 and STIL lesions compared to normal FTE. FBLN-5 and Emilin-2 were found higher expressed in p53 and to a lesser extent in STIL and were not detected in STIC lesions compared to normal FTE while SPTAN1 is higher expressed in some cells of the P53 and STIL lesions. CAVIN2 is absent of STIC lesions. (Fig. 5C, Supplementary Data 6).

**Table 2:** Immunoreactivity and prognostic effect of proteins overexpressed in p53 signature compared to normal tissue according to Protein Atlas.

Protein name	Immunoreactivity in FTE	Prognostic effect in OVC
CAVIN1	Not detected	Unfavorable***
SPTAN1	Moderate to strong positivity	Unfavorable*
FBLN5	Not detected	Unfavorable*
EIF3B	Strong positivity	Not significant
MOB1B	Moderate positivity	Not significant
EMILIN2	Moderate positivity	Not significant
SPTBN1	Moderate to strong positivity	Not significant
CAVIN2	Not detected	Not significant

Immunohistochemical staining of proteins in normal FTE tissue and prognostic effect of high expression of the corresponding gene in OVC were extracted from Human protein atlas (data credit: Human Protein Atlas available from v19.proteinatlas.org).

*p*-value is represented by stars ***p < 0.001; *p < 0.05 and no star for *p* > 0.05.

### Protein mutation

**Fig. 6:**
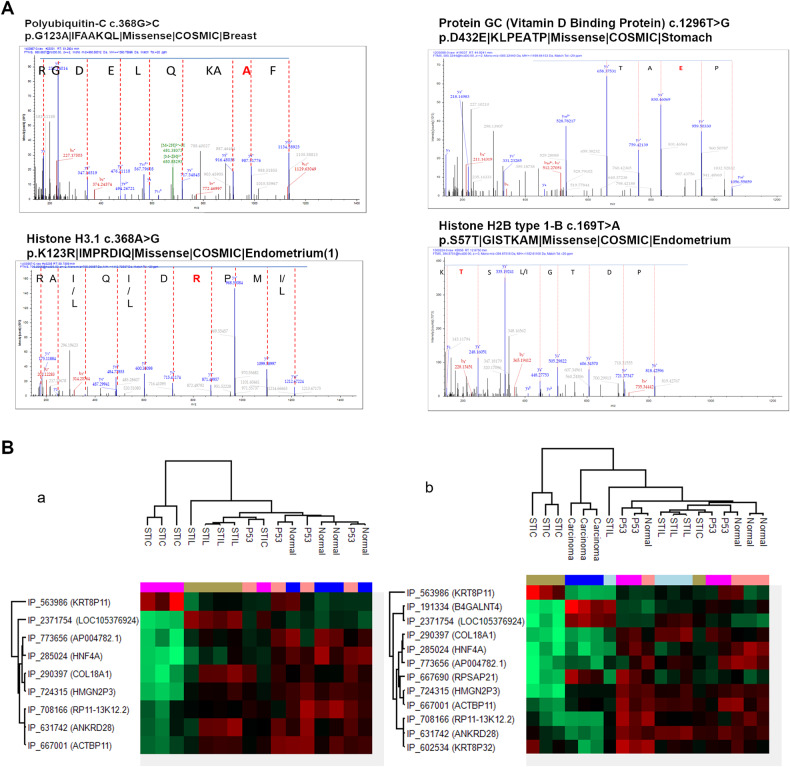
Potential mutations and Alternative proteins analysis. **A** Annotated MS/MS spectra showing the mutated amino acid in red. **B** Hierarchical clustering of the most variable alternative proteins between the lesion (a) or with adjunction of HGSC (b).

The linear relationship between the different preneoplastic lesions and HGSC has been suggested by the presence of genomic changes and mutations [50]. To identify specific modifications that may have occurred across the spectrum of the pre-cancerous lesions, we explored the presence of protein mutations in each lesion. For that purpose, we used the human database combined with the XMAn database [33]. This database contains information concerning mutated peptides that could be found in some cancers extracted from the COSMIC database. This database integration resulted in the identification of 83 peptide sequences containing possible mutations (Table 3, Supplementary Data 7). For four of them, it was possible to determine the amino acid modification directly via the tandem mass spectra (Fig. 6A). These peptides were derived from four proteins i.e., the vitamin D binding protein (GC), the polyubiquitin-C (UBC), the Histone H2B (H2B1C), and Histone H3.1 (H31). The mutations found in the protein GC (1296T>G p.D432E), lead to the sequence LP**E**ATPTELAK in the protein and have been identified in stomach cancer studies according to the COSMIC database. It was observed in data issued from two patients in normal tissue and a p53 signature lesion. Interestingly, this protein was found to be significantly under-represented in the STIC lesion (Supplementary Data 3). Similarly, the mutated peptide of the UBC was found in two normal tissues, two p53 signature lesions, and one STIL. The mutation (c.368G>C p.G123A), which leads to the new peptide the LIF**A**AKQLEDGR was previously identified in breast cancer. The mutated peptide (QVHPDTGIS**T**K) from Histone H2B (mutation c.169T>A p.S57T) was identified only in one normal tissue while the protein was not dysregulated (Supp. Data 3). Interestingly, this mutation has been identified before in the endometrium. The peptide of the histone H3.1 (VTIMP**R**DIQLAR) was observed in two normal tissue, one p53 signature, and one STIL lesion. This is downregulated in the STIC lesion (Supplementary Data 3). This mutation (c.368A>G p.K123R) has also been identified in the endometrium. By focusing on the identified 83 mutations (Table 3, Supplementary Data 7), it was observed that some were present in normal as well as in all lesion stages such as vimentin or collagen proteins. However, mutations on Histone H4, Histone H2B type 1-C/E/F/G/I are specific to the p53 signature, whereas mutation on 60S ribosomal protein L14 is specific to STIL, while mutation on (Na(+)/H(+) exchange regulatory cofactor NHE-RF1 differentiates STICs phenotype. Interestingly, in the transition from Normal to p53 signature, 7 mutated peptides have been identified. However, for p53 signature/STIL and Normal-p53/STIL, only one mutation has been observed respectively (on Isoform 2 of Tropomyosin beta chain and Histone H1.4 proteins). From normal- p53/STIL and STIC, 3 mutated peptides have been identified corresponding to Laminin subunit gamma 1, Vimentin, and Histone H3.1. It is of note that the last two mutations have also been detected in the endometrium cancer.

**Table 3:** Mutation detected in proteins from proteomic data obtained by spatially resolved proteomic using XMAn database.

Found in samples	Patients^a^ Number	Protein	Mutation	Cancers
p53 signature	2	Histone H4	c.239A>Gp.K80R|AKRRTVT|Missense	Endometrium
	2	Histone H2B type 1-C/E/F/G/I	c.181G>T p.G61C|KAMCIMN|Missense	Endometrium
STIL	3	60 S ribosomal protein L14	p.A159_K160insAA|AAAAAKVP|Insertion - in frame	Endometrium, Ovary, Breast
STIC	2	Na(+)/H(+) exchange regulatory cofactor NHE-RF1	c.767C>A p.P256H|GPLHVPF|Missense|	Large intestine
Normal-p53 signature	2/2	Hemoglobin subunit delta	c.427G>T p.A143S|NALSHKY|Missense	Lung
	2/2	Histone H2B type 1-C/E/F/G/I	c.133G>C p.V45L|VYKLLKQ|Missense	Breast
	2/2/1 HGSC	Actin, alpha cardiac muscle 1	c.166G>A p.V56I|DSYIGDE|Missense	Endometrium
	1/3	Synaptic vesicle membrane protein VAT-1 homolog	c.278T>G p.L93R|FADRMAR|Missense	Lung
	3/3	Isoform 2 of Drebrin-like protein	c.746G>A p.R249Q|SRNQNEQ|Missense	Endometrium
	2/3/1 STIL	Isoform 2 of Synaptopodin-2	c.3392C>T p.P1131L|RPTLWEA|Missense	Skin
	2/2/1 STIL	polyubiquitin c	c.368G>C p.G123A|IFAAKQL|Missense	Breast
p53signature/STIL	2/3	Isoform 2 of Tropomyosin beta chain	c.835C>A p.L279M|QTLMELN|Missense	Autonomic ganglia
Normal-p53/STIL	2/3/2:1 STIC	Histone H1.4	c.262G>T p.V88L|KSLLSKG|Missense	Esophagus
Nomal-p53/STIL/STIC	1/3/3/2/1 HGSC	Vimentin	c.1313C>G p.S438^a^|DTH|Nonsense	Endometrium
	4/4/3/2	Laminin subunit gamma-1	c.2033C>T p.P678L|SARLGPG|Missense	Breast
	4/4/4/4: HGSC	Histone H3.1	c.368A>G p.K123R|IMPRDIQ|Missense	Endometrium

^a^Only mutations found in more than two patients.

### Ghost Proteome

We next investigated the ghost proteome of STIC, STIL, p53 signature, HGSC and normal tissues. Ghost proteins are proteins translated from alternative open reading frames such as the untranslated regions of the mRNA (3’ and 5’UTR), from a shift in the open reading frame, or non-coding RNA (ncRNA). Using our raw proteomic data against the OpenProt database, 59 AltProts have been identified. More than 64% of these proteins are associated with sequences from non-coding RNA (ncRNA) and from the mRNA coding for RefProt. 23% are from 5’UTR, 33% from the 3’UTR, and 42% from a shift in the CDS (Supp. Data 8). Among these proteins (Table 4, Supplementary. Data 8), we identified IP_270787 (AltZNF709) in normal tissues. IP_2326985 is issued from an ncRNA (LOC105376003) and is only found in STIC. The AltProt IP_232994 (AltMYEF2), coded by the 3’UTR part of the transcript mRNA referenced to translate the MYEF2, is identified only in HGSC samples. IP_572777 (from the ncRNA: SDR16C6P), IP_774783 (from the ncRNA: RP11-358N4.6) and IP_285024 (from the ncRNA: HNF4A) are specific to Normal, p53 signature and STIL samples. In both Normal/p53/STIL and STIC samples, 17 AltProts were identified (Table 4). Using the ANOVA test, 12 AltProts displayed significant variations in the different lesion stages. Nine AltProts are derived from ncRNA, one from 3’UTR, and two from a shift in the CDS. The hierarchical clustering and heatmap representation (Fig. 6Ba) revealed a clear separation between STIC and the other lesions. The second branch separates STIL from the normal-p53 signature. The pseudogene KRT8P11 (IP_563986) is overexpressed in STIC (Fig. 6Ba), whereas COL18A1 (IP_290397) pseudogene is overexpressed mainly in the STIL samples and slightly in P53 and is underexpressed in carcinoma whereas LOC105376924 is present in STIL and in carcinoma and absent in P53 samples (Fig. 6Bb). The other AltProts are differentially expressed in p53 lesions and Normal tissue but are significantly down-regulated in the STIC or non-expressed in STIL (Fig. 6Ba). When data from HGSC were added, the separation of STIC from other samples was observed, carcinoma samples composed the second branch, and a third branch is divided into subbranches. These subdivisions group included three of the fourth STIL samples and three of the fourth Normal samples (Fig. 6Bb). Based on our findings, it is proposed that KRTP8P11 (IP_563986) is a specific marker of STIC, B4GALN (IP_191334) is specific of HGSC, and KRT8P32 (IP_602534) is overexpressed in two p53 signature samples and one of the normal tissue samples but also highly decreased in HGSC. Ultimately, COL18A1 (IP_290397) is specific to STIL. The other AltProts were observed between Normal-p53 signatures with most of them significantly down-regulated in STIC (Fig. 6Bb).

**Table 4:** Summary of the repartition to the Venn diagram result of the 45 AltProts identified.

Altprot	Normal	P53	STIL	STIC	Carcinoma
IP_270787	X				
IP_572777	X	X	X		
IP_774783	X	X	X		
IP_285024	X	X	X		
IP_669028	X	X	X	X	
IP_563986	X	X	X	X	
IP_621829	X	X	X	X	
IP_595290	X	X	X	X	
IP_730500	X	X	X	X	
IP_2322038	X	X	X	X	
IP_763981	X	X	X	X	
IP_708166	X	X	X	X	
IP_737172	X	X	X	X	
IP_290397	X	X	X	X	
IP_652773	X	X	X	X	
IP_689518	X	X	X	X	
IP_721145	X	X	X	X	
IP_060976	X	X	X	X	
IP_632428	X	X	X	X	
IP_274301	X	X	X	X	
IP_138273	X	X	X	X	
IP_716829	X		X	X	
IP_2326985				X	
IP_591310		X	X	X	X
IP_2285716	X		X	X	X
IP_572435	X	X		X	X
IP_2323408	X			X	X
IP_079312		X	X		X
IP_667690		X	X		X
IP_076499		X	X		X
IP_601870	X		X		X
IP_755940	X	X			X
IP_232994					X
IP_602534	X	X	X	X	X
IP_667001	X	X	X	X	X
IP_755869	X	X	X	X	X
IP_658355	X	X	X	X	X
IP_724315	X	X	X	X	X
IP_591792	X	X	X	X	X
IP_631742	X	X	X	X	X
IP_688853	X	X	X	X	X
IP_2371754	X	X	X	X	X
IP_191334	X	X	X	X	X
IP_773656	X	X	X	X	X
IP_794359	X	X	X	X	X

14 AltProt have been deleted because they are identified but without a sufficient abundance to be quantified. The AltProt identification accession ID is according to the OpenProt Database. Venn diagram representation is based on the abundance obtain after analysis, function to the triplicate samples type (normal, p53, STIL, STIC, Carcinoma).

### SpiderMass analysis

**Fig. 7:**
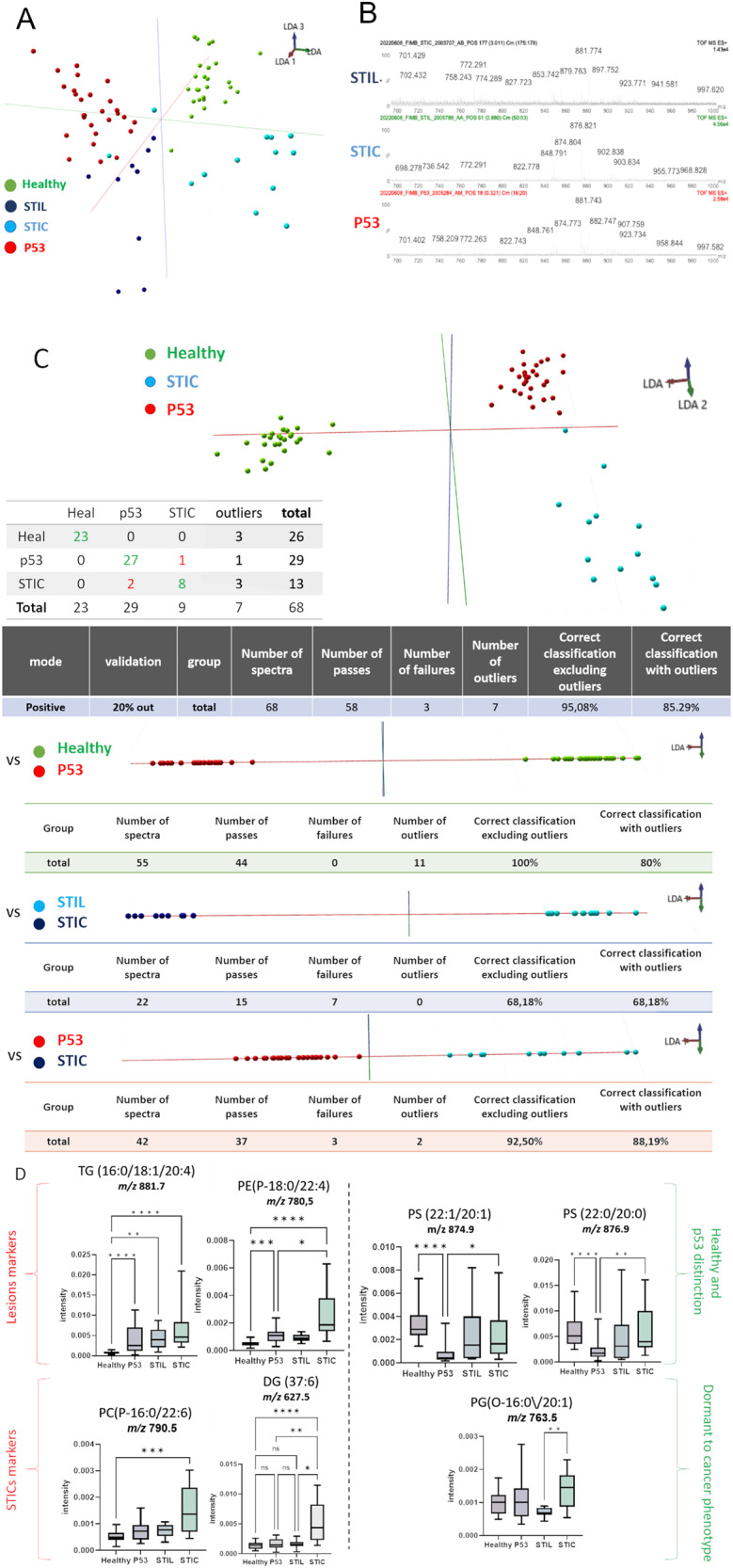
SpiderMass ex vivo real-time analyses of tissues section. **A** Mass spectra obtained by SpiderMass from P52, STIL, and STIC preneoplasia lesion in fimbria, **B** The built PCA-LDA classification model based on 3 preneoplasia lesion stages; P53, STIL and STIC. **C** LDA representation of the 3-class PCA-LDA (right). The table (right) represents the “leave-one-patient-out” cross-validation results of the built classification model. **D** Specific markers detected in P53, or STIC. (****p < 0.0001, ***p < 0.001, **p < 0.01, *p < 0.05) no star for *p* > 0.05).

To validate the classification obtained by MALDI-MSI, we analyzed samples with SpiderMass technology. Following the acquisition of the MS spectra in positive ion mode, a PCA analysis of the generated spectra acquired from healthy, p53 signature, STIL and STIC tissues was performed. The PCA features were subjected to a supervised analysis using linear discriminant analysis (LDA) [51] which resulted in 4 different groups (Fig. 7A). According to Fig. 7A, LDA 1 discriminated the Healthy group from the p53 and STIC groups. However, p53 signature are less separated from STIL than from Healthy. This was also observed when we compared spectra. The p53 signature and STIL spectra are much closer than STIC ones (Fig. 7B). Cross-validation results obtained from the p53 signature, Healthy and STIC groups using the “20% out method” shows excellent classification rates with 95.08% and 85.29% without and with outliers respectively. An excellent classification between P53 and Healthy with 100 and 80% including or not outliers respectively showed significant discrimination of both classes taken from the same tissue section. A correct separation of 75% with and 72.93% without outliers between P53 and STIL. Finally, a better classification rate of 92.5% and 88.1% with and without outliers was performed between P53 and STIC (Fig. 7C).

Some examples of discriminant ions (m/z) between the four groups corresponding to lipids, are presented with their normalized intensities in (Fig. 7D) and were identified by MS/MS. Thanks to the generated boxplot we were able to find lesions’ lipidic markers such as TG (16:0/18:1/20:4) (881.7 *m/z*) or PE (P-18:0/22:4) (780.5 *m/z*) that are underexpressed in healthy tissues. In addition to these 2 lipids, the overexpression of PC(P-16:0/22:6) (790.5 *m/z*) and DG (37:6) (627.5 *m/z*) in STICs enables the differentiation between healthy and lesion phenotypes from the cancerous one. Interestingly, we were able to discriminate p53 lesion from healthy tissue by PS (22:1/20:1) (874.9 *m/z*) and PS (22:0/20:0) (876.9 *m/z*) which are underrepresented in a p53 lesion. Finally, we were able to distinguish STILs from STICs by the PG(O-16:0/20:1) (763.5 *m/z*). In fact, this lipid is underrepresented in STILs compared with other phenotypes.

## Discussion

High-grade serous ovarian carcinoma (HGSC) may originate at the tubal-peritoneal junction where the tubal fimbriae’s specialized epithelium meets the peritoneum covering the serosal surface of the fallopian tube, according to recent theories [20, 52]. This region represents a cancer hotspot due to the transition between different types of epithelia, and there is evidence supporting the presence of cancer-prone stem cells in this area. STIC (serous tubal intraepithelial carcinoma) has emerged as a precursor lesion recognized by its preferential localization in the fimbriae end of the fallopian tube, which has led to the recommendation of standardized and detailed sampling of tubal specimens using the SEE-FIM protocol. However, diagnosing STIC based on morphology alone has low reproducibility. The histological examination of fallopian tubes has revealed a variety of pre-neoplastic lesions, including p53 signature and STIL (serous tubal intraepithelial lesion) lesions, which have not been previously characterized. The temporal relationship between these lesions and HGSCs is still debated.

It should be noted that pre-neoplastic lesions of the FTE are not commonly used for cancer detection or diagnosis in clinical practice, as they are not easily detectable. Although the goal of our study was not to identify diagnostic biomarkers due to the limited number of patients, we aimed to provide a comprehensive description of the proteomic content of pre-neoplastic lesions in BRCA1 mutation carriers. This description can help to better understand the transition between different lesion types and their potential link to ovarian cancer. Future studies with larger cohorts are necessary to identify potential biomarkers for early detection of these lesions. Our study provides the first in-depth proteomics and system biology analysis of epithelial cells in the fimbria end of the fallopian tube to better understand the different pre-neoplastic lesions. The robust and unique protocol established for tissue section sampling provides a strong foundation for future research in this area. We developed a novel strategy based on pathology routine protocol to investigate pre-cancerous lesions. Obtaining several tissue sections containing these lesions is challenging due to their limited cell numbers. The SEE-FIM protocol allows for their localization and differentiation but on a limited number of slides. To better understand the transitions of the lesions, we performed IHC-guided spatially resolved proteomic analyses on each identified lesion. Our workflow is compatible with proteomic analysis, allowing for targeted digestion of each region highlighted by IHC. The tissue section surface recovery protocol does not alter the proteomic content and enables an accurate comparison of quantified proteins between each lesion. Our protocol has the advantage of reusing the diagnostic IHC slides, which provide the localization and categorization of each lesion. It allows for the in-depth study of pre-cancerous lesions using a limited number of tissue sections.

We initially verified, using MALDI-MSI, that there are discernible differences in the proteomic content of pre-neoplastic lesions. With this information, we performed a spatially resolved proteomics analysis of each lesion in comparison to normal tissue. By comparing the proteomic content of HGSC and pre-neoplastic lesions found in the fallopian tube, we observed intriguing groupings that may correspond to the chronology of HGSC development proposed by different studies. Our analysis revealed that the normal tissue and p53 signature lesions cluster together and are near the STIL. In contrast, the STIC lesion presents a distinct proteomic signature and is closer to HGSC than the other lesions. Given the differences in composition between the fallopian tube tissue and the ovary, we focused our analysis on the pre-neoplastic lesions to gain a deeper understanding of the biological processes involved in their molecular transition.

By comparing the global content of each lesion, we successfully identified specific markers for p53 signature, the protein RNA-binding protein 10 (RBM10)- 3 specific proteins in STIC lesions -i.e., Glucosamine (N-acetyl)-6-sulfatase (GNS), Upstream binding transcription factor, RNA polymerase I (UBTF) and ATPase family, AAA domain containing 3 (ATAD3A/B). Specific mutated proteins from each lesion stage were also characterized. These mutations (mutated Histone H4, Histone H2B type 1-C/E/F/G/I) included in p53 signature, mutated 60 S ribosomal protein L14 in STIL, and mutated (Na(+)/H(+) exchange regulatory cofactor NHERF1 in STIC).

The p53 signature lesion is only characterized by a limited number of epithelial cells (~10) presenting a p53 overexpression. In our analysis, the p53 signature samples are close in their protein content to the normal tissue. Nevertheless, some specific proteins of these lesions could be obtained. Indeed, a signature emerged and demonstrated that among the 8 proteins overexpressed in the p53 signature, some of them are linked with a poor prognosis as follows: i) CAVIN1, SPTAN1 and FBLN5 for OC; ii) (EIF3B) for renal, head and neck cancers iii) MOB1B, EMILIN-2, EIF3B for liver cancer. On the contrary, 3 are linked with a favorable prognosis for renal cancer (SPTAN1, SPTBN1, CAVIN2).

We also dissected the underlying molecular mechanisms. In addition, the whole proteome analysis showed that the transition from normal to p53 signature was characterized by a residual immune response, translation activation, regulation of trafficking through cavins proteins, and ECM modifications via emilins.

From p53 signature to STIL, we showed that upregulated proteins were involved in the adhesion and anchoring of cells in the ECM, allowing stabilization of cells, along with an increase in maintenance activity instead of cell proliferation. Immune response and inflammation processes are still maintained, but an overall decrease of all metabolic processes, especially the TCA cycle, is observed without any Warburg effect. These observations are in line with a recent study suggesting that STIL could be considered “Dormant STICs” and take a prolonged time of more than one decade to develop into STIC [21]. We observed that mainly cell growth and maintenance metabolism pathways are downregulated in STIL, which could explain this “dormant” characteristic.

In contrast to the dormant profile of STILs, STICs show a more aggressive dysregulation with a decrease in the protein involved in cell adhesion. This could reflect the start of a cancer invasion step. STIC’s upregulated proteins show various functions inside the cells and appear to be involved in different steps of cancer processes. An overexpression of proteins involved in energy pathways is observed. If we compare the molecular functions involved in the three different groups, calcium-dependent protein binding is overrepresented. In addition, heat shock protein activity is highly represented in this dataset. Moreover, proteins involved in telomere activities and centrosome maturation are specific to STICs compared to other lesions showing their cancerous specificity. Interestingly, these two processes are linked with early tumorigenesis [53], and STIC precedes the development of many HGSCs [54]. A low expression of proteins involved in the extracellular structure organization and cell adhesion molecule activity was observed in STIC lesions. This would then suggest a breaching of the basement membrane and an increase in cell mobility that could induce an escape of these pre-cancerous cells to a distant organ. Finally, we observed a high level of proteins involved in extracellular vesicles including exosome production which could consequently modify the microenvironment and promote distant carcinogenic processes.

From pre-neoplastic lesions to HGSC, one protein was found common in the pre-neoplastic lesions and HGSC, the Fibrillarin (FBL) protein. FBL is one of the core proteins of box C/D small nucleolar ribonucleoprotein complexes (snoRNP). This complex is involved in the first steps of pre-rRNA processing [55]. It has also been shown that dysregulation of ribosome biogenesis plays key roles in oncogenesis [56]. Overactivation in cancer cells of ribosome biogenesis could be due to a loss of function of RNA polymerase repressors such as p53 [57]. Particularly FBL expression has been demonstrated to be correlated with p53 activity [58]. High levels of FBL protein were associated with the expression of mutant p53 and contributed to tumorigenesis by altering translational control of key cancer genes. UBTF is only present in STIC and HGSC. This protein is known to be a transcription factor involved in the regulation of the RNA polymerase I (Pol I), implicated in the regulation of cell cycle checkpoint, DNA damage response and regulated by MYC [59, 60]. Different studies have shown a link between Pol I initiation factor and chemoresistance of ovarian cancer [61].

Similarities between these pre-neoplastic lesions and HGSC have been found especially concerning *TP53* mutations and other genomic changes, but without excessive cell proliferation [62]. In that context, we decided to explore the presence of mutations at the protein level in the different steps of tubal tumor development. The use of a specific database for protein mutation, XMAn, resulted in the identification of 83 mutated peptide sequences, of which 4 were observed directly in the tandem mass spectra. It is interesting to observe that most of the proteins mutated in the p53 signature have been initially identified in endometrium cancer and are linked to histones and ribosomes suggesting that epigenetic regulation may be involved in the translation of the pre-neoplastic lesions.

Polyubiquitin C is related to proteasome and self-antigen presentation. Synaptopodin-2 and the Isoform 2 of Drebrin-like protein are actin-binding proteins known to be respectively invasive cancer biomarkers [63] and potential markers for breast, lung, and colorectal cancers [64]. Finally, Actin, alpha cardiac muscle 1 is involved in cisplatin ovarian cancer cell resistance [65] and the synaptic vesicle membrane protein VAT-1 homolog is a marker of epithelial cells [66].

These mutated proteins were only found in histones from STIL and STIC. Most of the mutated peptides observed in STIL, STIC, p53/STIL, or in Normal/p53 signature/STIL/STIC are linked to the cytoskeleton and cell migration. These mutations could be key drivers of cancer’s process from the epigenetic modification step, and then on cytoskeleton modifications for cell migration and cancer development.

Using a dedicated database, identification of significantly variable AltProts, provides us with a new view of unknown markers. Some of the identified AltProts or transcripts in the present work have been described in other cancer studies. For example, the pseudogene KRT8P32 has been identified in breast cancer [67] and the pseudogene ACTBP11 was observed in GM12878 (B cells) cell lines according to Diana-LncBase V3 [68]. Additionally, it has recently been shown that AltANKRD28 can act as a novel BRCA1-interacting protein in breast and ovarian cancer [69]. Furthermore, RP11-13K12.2 is described to be overexpressed in pediatric ovarian fibrosarcoma [70]. COL18A1 and B4GALNT4 are also overexpressed in endometrial and ovarian cancers according to the HPA and are unfavorable markers for overall survival for both cancers. Some of these AltProts are specific to the type of lesion - i.e. LOC105376924 and COL18A1 in STILL, KRT8P11 in STIC, and ANKRD28 in STIL-STIC. Altogether, these Altprots may be clinically relevant, and further investigation may improve the understanding of the disease.

Finally, our results identified AltProts and many mutations affecting histones and cytoskeleton proteins observed between normal to p53 signatures compared to the other lesions. These findings are in line with the hypothesis of epigenetic reprogramming toward carcinogenesis and cell transformation [71]. A recent study established that epigenetic reprogramming occurs specifically in the proximal end of the fallopian tubes in *BRCA* mutation carriers. This epigenetic reprogramming event is driven by aberrantly high AICDA (also named AID, activation-induced cytosine deaminase) expression and is an integral early pre-malignant event in HGSC development. In this context, our results would provide an interesting starting point for further studies, especially concerning the potential link to the endometrial carcinoma cells’ exfoliation due to epigenetic factors leading to carcinogenesis [6]. It is also hypothesized that STIL represents exfoliated precursor cells that eventually undergo malignant transformation within the peritoneal cavity [72].

Using SpiderMass technology on FFPE tissue, we have successfully demonstrated the ability to distinguish healthy tissues from pre-neoplastic conditions in a lipidomic study. Specifically, we identified two phosphatidylserines (PS (22:1/20:1) and PS (22:0/20:0)) as discriminatory markers between p53 signature and healthy tissue, while PE(P-18:0/22:4) and TG (16:0/18:1/20:4) were found to be overexpressed in the lesions. Additionally, we specifically observed the presence of phosphatidylcholine (P-16:0/22:6) and diglyceride (DG (37:6)) in STICs, enabling their distinct identification. Notably, the inclusion of phosphatidylglycerol (PG (O-16:0/20:1)) allowed us to differentiate between STILs and STICs. This significant variability in lipid composition distinguishes our study from previous investigations in ovarian cancer. In Ovarian Clear Cell Carcinoma (OCCC) studied from FFPE tissues, the number of unsaturated lipid species increases whereas the number of saturated lipid species decreases in OCCC compared to the controls. PE (32:1), PE (34:1), PI (34:1), and PS (36:1), present a common signature in OCCC [73]. In serous ovarian cancer, the overexpression of fatty acid binding protein 4 leads to an increase of glycerolipids, glycerophosphoethanolamines, glycerophosphoinositols such as LysoPE, LysoPG, and LysoPI [74]. Cancer cells rely on lipogenesis to adapt to cytotoxic stress in the tumor microenvironment. This is particularly important in tumor areas where the exogenous supply of fatty acids is scarce, such as in hypovascular and hypoxic regions. Moreover, it is also interesting to note the presence of several dietary fatty acid (FA) precursors (*e.g*., 20:4, 22:4) issued from omega-6 FA to give polyunsaturated FA known to enhance the carcinogenic process in different cancers [75]. The dietary FA are used by cancer cells as energy supply by lipogenesis for membrane biosynthesis, signaling processes and ROS process inhibition. The observations we made regarding lipid modulation are substantiated by the presence of several dysregulated metabolic enzymes in cancer. Notably, we found that PHGDH is overexpressed in STIL and STIC lesions, which is consistent with its high expression in other cancer types, including breast, colon, and endometrial cancer. Moreover, there is evidence linking triglyceride levels to PHGDH DNA methylation, suggesting a potential regulatory role of this enzyme in triglyceride metabolism in cancer [76, 77]. Furthermore, our study identified other proteins related to lipid metabolism that are overexpressed in STICs, such as MARCKS-related protein, 2,4-dienoyl-CoA reductase [78], Trifunctional enzyme subunit beta [79], and 3-ketoacyl-CoA thiolase. Conversely, certain lipid metabolism proteins, like Aldehyde dehydrogenase family 3 member B1 (ALDH3B1), implicated in the oxidation of medium and long-chain lipid-derived aldehydes generated in the plasma membrane [80], were found to be underexpressed in STILs. Finally, the study of Ackerman et al. [81] shows the importance of saturated fatty acid storage into triglycerides to prevent toxicity and preserve homeostasis in a hypoxic environment on clear cell renal cell carcinoma by releasing oleate (18:1) that will embed into phospholipids. Interestingly, TG (16:0/18:1/20:4) was found discriminative of fimbria lesions and even more abundant in STICs. STIC is a high-proliferative lesion that exhibits the potential to utilize triglycerides as a means to safeguard its homeostasis during growth. Furthermore, it has been observed that patients with BRCA1 and BRCA2 mutations demonstrate elevated levels of triglycerides [82]. This finding implies that individuals with BRCA1 and BRCA2 mutations may have heightened triglyceride levels, which could contribute to the enhanced survivability of lesional cells by preserving homeostasis and facilitating the development of STICs.

## Conclusion

In conclusion, this study provides valuable insights into the molecular events that lead to the development of ovarian cancer. By analyzing the proteomic content of pre-neoplastic lesions in the fimbriated end of the fallopian tube, we were able to identify the underlying mechanisms and potential timeline of events that lead to the formation of ovarian cancer. Our findings support previous studies proposing a sequence of molecular events that start with p53 signature lesions and progress through STIL to STIC and ultimately to HGSC. The results of this study contribute to a better understanding of the etiology of ovarian cancer and may have important implications for the development of early-detection biomarkers and therapeutic strategies.

### Supplementary information

Supporting information

AJ-checkckist

Supp Data 1

Supp Data 2

Supp data 3

Supp Data 4

Supp data 5

supp Data 6

Supp data 7

Supp Data 8

## Data Availability

The MS data sets and Perseus result files used for analysis were deposited at the ProteomeXchange Consortium (http://proteomecentral.proteomexchange.org, [83]) via the PRIDE partner repository with the data set identifier PXD020024 (for reviewer access only, Username: reviewer86596@ebi.ac.uk; Password: x88jqaDV).
