# Supplementary material for: Fallopian tube lesions as potential precursors of early ovarian cancer: a comprehensive proteomic analysis
Source: Cell Death Dis. 2023 Sep 30;14(9):644. doi: 10.1038/s41419-023-06165-5 (PMC10541450; doi:10.1038/s41419-023-06165-5)
Supplement: Supplementary file 1 — Supporting information [file 41419_2023_6165_MOESM1_ESM.docx]

**SUPPORTING INFORMATION**

**Fallopian tube lesions as potential precursors of early ovarian cancer: A comprehensive proteomic analysis.**

Maxence Wisztorski^1*^, Soulaimane Aboulouard^1*^, Lucas Roussel^1*^, Marie Duhamel^1*^, Philippe Saudemont^1^, Tristan Cardon^1^, Fabrice Narducci,^1,2^, Yves-Marie Robin^1,2^, Anne-Sophie Lemaire^1,2^, Delphine Bertin ^1,2^, Nawale Hajjaji^1,3^, Firas Kobeissy^4,5^, Eric Leblanc^1,2ǂ^, Isabelle Fournier^1,6ǂ*^, Michel Salzet^1,6ǂ*^

^1^Univ.Lille, Inserm, CHU Lille, U-1192 – Laboratoire Protéomique Réponse Inflammatoire Spectrométrie de Masse - PRISM, F-59000 Lille, France

^2^ Department of Gynecology Oncology, Oscar Lambret Cancer Center, 59020 Lille, France

^3^ Medical Oncology Department, Oscar Lambret Cancer Center, 59020 Lille, France

^4^ Department of Neurobiology, Center for Neurotrauma, Multiomics & Biomarkers (CNMB), MorehouseSchool of Medicine, GA 30310 Atlanta, GA, United States

^5^ Department of Biochemistry and Molecular Genetics, Faculty of Medicine, American University of Beirut, Beirut, Lebanon.

^6^ Institut Universitaire de France, 75000 Paris

*Equivalent contribution, Co first author

**^ǂ^** Co-Corresponding author, Co Last author

**TABLE OF CONTENTS**

**Supplementary files:**

**Supp. Data 1:** List of quantified proteins corresponding to Venn diagram for lesions comparison

**Supp. Data 2:** List of quantified proteins corresponding to Venn diagram for lesions and HGSC comparison

**Supp. Data 3:** List of quantified proteins significantly modified in the different preneoplastic lesions

**Supp. Data 4:** Significantly enriched pathways obtained by enrichment analysis using Panther Db

**Supp. Data 5:** Quantitative comparison of proteins modified between normal tissue and p53 signature

**Supp. Data 6** : Immunohistochemistry experiments performed by fluorescence based on the specific markers (CAVIN1,CAVIN2, EIF3B, SPTAN1) identified from the different neoplastic lesion compared to healthy tissues.

**Supp. Data 7:** List of mutated peptides identified using the XMAn database

**Supp. Data 8:** List of alternatives proteins identified

**Supplementary figures:**

**Figure S1:** Mass Spectrometry Imaging of different preneoplastic lesions.

**Figure S1:** Visualization of protein levels in the different lesions for proteins known to be involved in the Warburg effect.

**Supplementary Methods:**

Mass spectrometry Imaging and data analysis


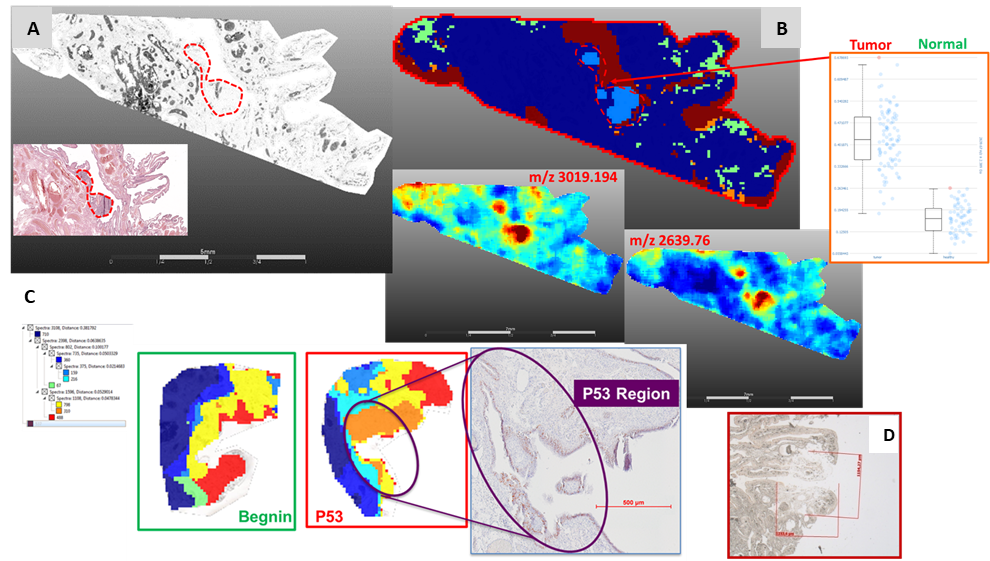


**Figure S1: Mass Spectrometry Imaging of different preneoplastic lesions.** A) Optical visualization of a tissue section showing a highlighted region with a STIC. B) Spatial segmentation was used to identify and visualize regions with different molecular signatures. The image shows the extracted ions with m/z 3019.194 and m/z 2636.76. C) Spatial segmentation comparison between normal tissue and a tissue section with a region presenting a p53 signature. D) Example of a lesion region subjected to trypsin digestion and liquid microextraction, with the resulting visualization.


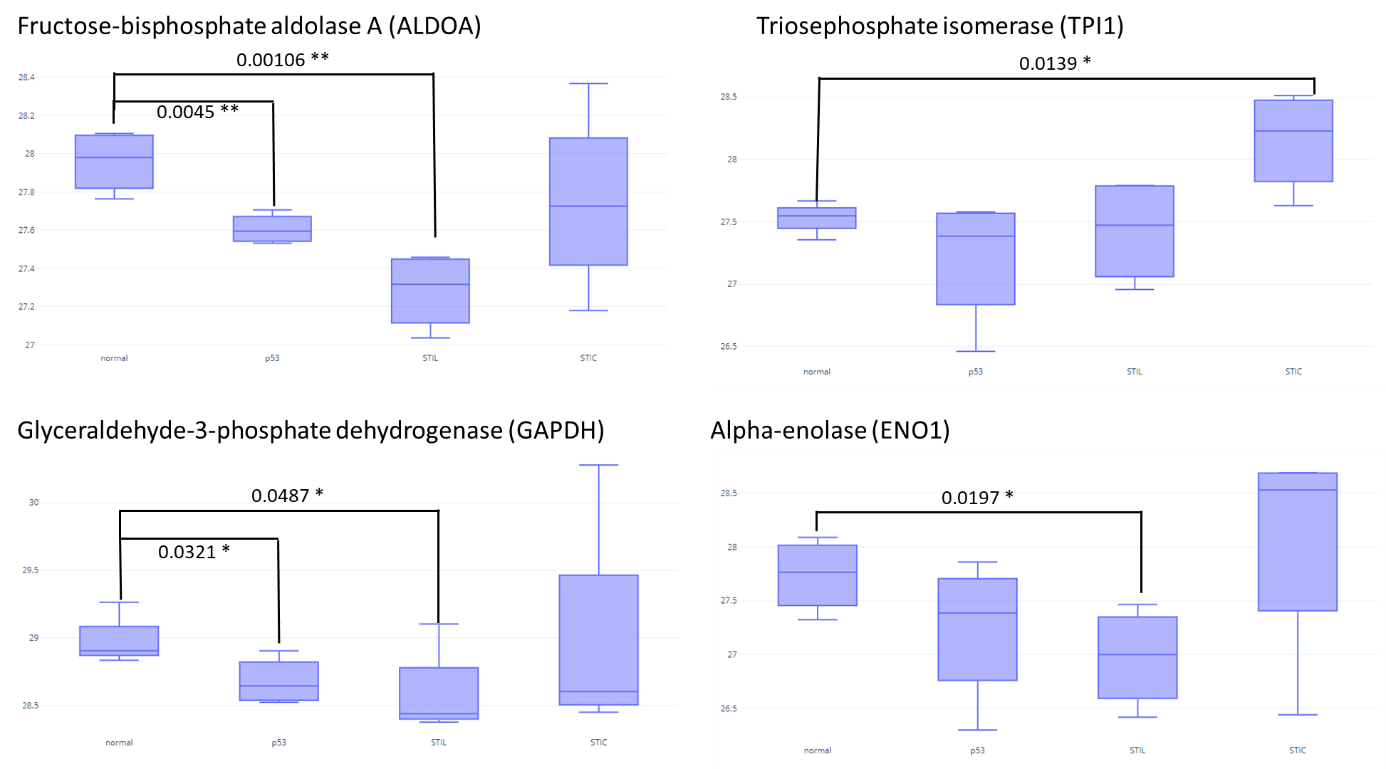


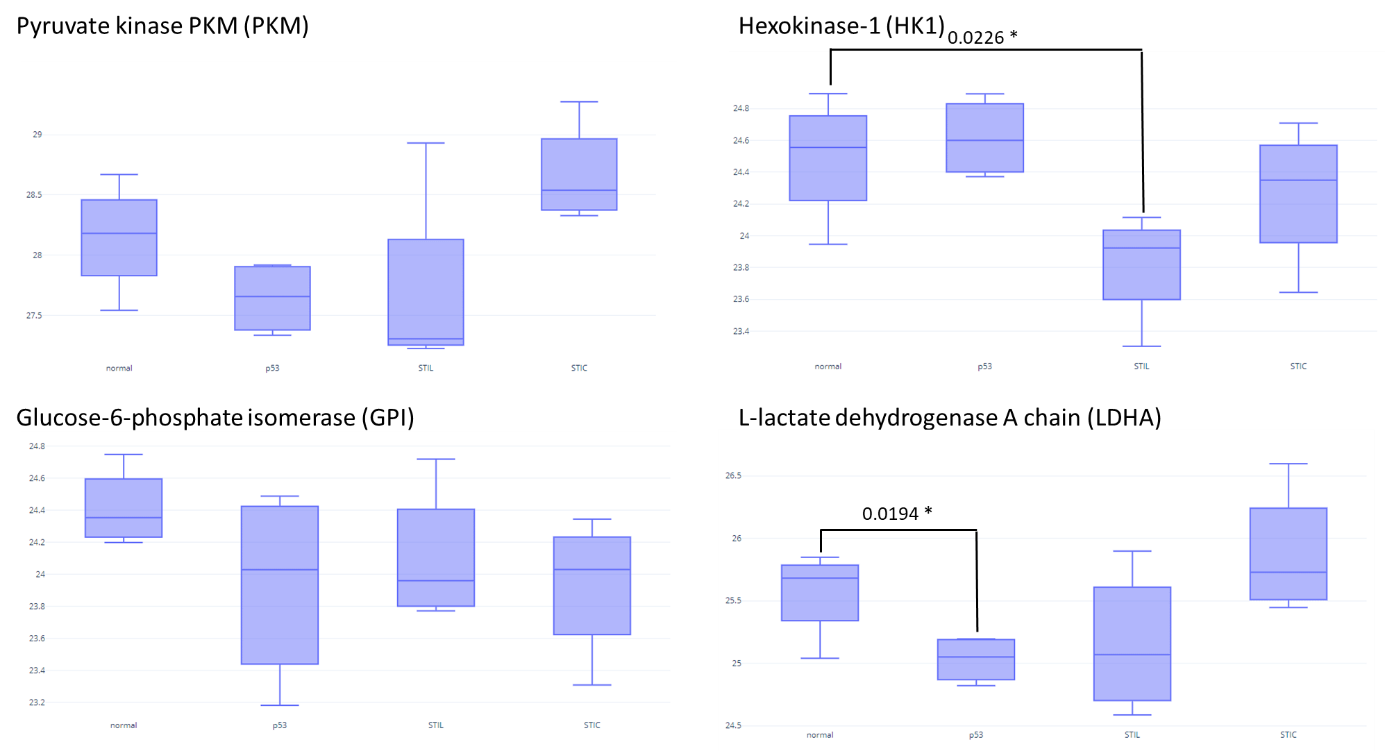


Figure S2: **Visualization of protein levels in the different lesions for proteins known to be involved in the Warburg effect.** Log(LFQ) values were used for the representation of Fructose-bisphosphate aldolase A (ALDOA), Triosephosphate isomerase (TPI1), Glyceraldehyde-3-phosphate dehydrogenase (GAPDH), Alpha-enolase (ENO1), Pyruvate kinase PKM (PKM), Hexokinase-1 (HK1), Glucose-6-phosphate isomerase (GPI) and L-lactate dehydrogenase A chain (LDHA). A t-test is used to compare the value between normal tissue and each lesion. P-value is represented by stars (*** p < 0.001, ** p < 0.01 , * p < 0.05 no star for p > 0.05).

**Supplementary Methods:**

**MALDI mass spectrometry imaging (MALDI MSI)**

In this study, 7 µm-thick FFPE tissue sections were used for MALDI MSI analysis, following previously established protocols (Fournier et al., 2003; Lemaire et al., 2007; Lemaire et al., 2006a; Lemaire et al., 2006b; Wisztorski et al., 2007). To ensure optimal trypsin digestion, a trypsin solution (60 μg/mL in NH4HCO3 50 mM) was uniformly sprayed onto the tissue surface for 15 minutes using an electrospray nebulizer connected to a syringe pump (flow rate 180 nL/min). The incubation chamber, ImagePrep (Bruker Daltonics, Bremen, Germany), was used to microspray water heated to 37°C for 2 hours with a constant humidity atmosphere maintained by filling a small container with 95°C water. After digestion, a solid ionic matrix, HCCA/ANI (Lemaire et al., 2006), was deposited using ImagePrep, where 36 μL of aniline were added to 5 mL of a solution of 10 mg/mL HCCA dissolved in ACN/0.1% TFA aqueous (7:3, v/v). A uniform layer of matrix was achieved by monitoring scattered light in real-time. MALDI MSI experiments were performed on an Ultraflex II MALDI-TOF/TOF instrument (Bruker) with a smartbeam II solid state laser, acquiring mass spectra in positive reflector mode between 800-4000 m/z range. Spectra were recorded by averaging 400 laser shots per pixel, with a 70 μm spatial resolution raster and a laser repletion rate of 200Hz.

**MALDI MSI data processing and analysis**

The MALDI-MSI data were analyzed using SCiLS Lab software (SCiLS Lab 2019, SCiLS GmbH) in accordance with standard processing methods for MALDI MSI. First, a convolution method was used to remove the baseline and then the data were normalized using the Total Ion Count (TIC) method (Klein et al., 2014; Trede et al., 2012). Next, the bisecting k-means algorithm (Alexandrov et al., 2010) was employed to cluster the pre-processed data, resulting in a spatial segmentation.

To minimize pixel-to-pixel variability, the clusters were subjected to edge-preserving image denoising and arbitrarily assigned a color. It should be noted that multiple disconnected regions may have the same color, indicating similar molecular content. The resulting spatial segmentation maps were displayed as a dendrogram generated through hierarchical clustering, with the dendrogram branches determined by a distance calculation between each cluster. Selection of different dendrogram branches enabled the differentiation and color-coding of distinct molecular composition regions.

**NanoLC-ESI-MS²**.

After the liquid extraction, samples were freeze-dried in a SpeedVac concentrator (SPD131DPA, Thermo Scientific, Waltham, Massachusetts, USA), reconstituted with 10µL 0.1% TFA and subjected to solid-phase extraction to remove salts and concentrate the peptides. This was done using a C-18 Ziptip (Millipore, Saint-Quentin-en-Yvelines, France), eluted by an ACN/0.1% TFA (8:2, v/v) and then the samples were dried for storage. Before analysis, samples were suspended in 20µL ACN/0.1% FA (2:98, v/v), deposited in vials, and 10µLs were injected for analysis. The separation, prior to MS, used online reversed-phase chromatography realized with a Proxeon Easy-nLC-1000 system (Thermo Scientific) equipped with an Acclaim PepMap trap column (75 μm ID x 2 cm, Thermo Scientific) and C18 packed tip Acclaim PepMap RSLC column (75 μm ID x 50 cm, Thermo Scientific). Peptides were separated using an increasing amount of acetonitrile (5%-40% over 140 minutes) with a flow rate of 300 nL/min. The LC eluent was electrosprayed directly from the analytical column and a voltage of 2 kV was applied via the liquid junction of the nanospray source.

The chromatography system was coupled to a Thermo Scientific Q-Exactive mass spectrometer. The mass spectrometer was programmed to acquire in a data-dependent mode for the 10 most intense peaks. The survey scans were acquired in the Orbitrap mass analyzer operated at 70,000 (FWHM) resolving power. A mass range of 200 to 2000 m/z and a target of 3E6 ions were used for the survey scans. The MSMS analysis was performed using HCD with a normalized collision energy of 30 eV, a mass range between 200 to 2000, an AGC of 5e4 ions, a maximum injection time of 60ms and a resolution set at 17,500 FWHM. The method was set to analyze the top 10 most intense ions from the survey scan and dynamic exclusion was enabled for 20 s.

**Spidermass Technology :**

The global design of the SpiderMass instrument setup has been described in detail elsewhere^49^. Briefly, the system is composed of three parts including a laser system for micro-sampling of tissues which is set remotely, a transfer line allowing for transfer of the micro-sampled material to the third part, which is the mass spectrometer itself ^50^. The first part is composed of a tunable wavelength OPO which is tunable from 2.8 µm to 3.1 µm (Radiant version 1.0.1, OPOTEK Inc., Carlsbad, CA, USA) pumped by a pulsed Nd:YAG laser (pulse duration: 5 ns, λ=1064 nm, Quantel, Les Ulis, France). A biocompatible laser fiber (450 µm inner diameter; length of 1 m; Infrared Fiber Systems, Silver Spring, CO, USA) is connected to the laser system output and a handpiece including a 4 cm focusing lens is attached to the end of the laser fiber. The handpiece with a 4 cm focusing lens allows the user to hold the system and screen the surface of raw tissues at a resolution of 400 µm. In these experiments the irradiation time was fixed to 10 sec at 4 mJ/pulse laser energy corresponding to a laser fluence of ~3 J/cm2. The laser energy was measured at the focal point of the focusing lens using a power meter (ThorLabs, Maisons-Laffitte, France). The second part of the system corresponds to a 3-meter length transfer line made from a Tygon ND 100-65 tubing (2.4 mm inner diameter, 4 mm outer diameter, Akron, USA). The transfer line is attached on one side onto the laser hand piece at the end of the laser fiber and on its other side directly connected to the mass spectrometer (Xevo, Waters, Manchester, United Kingdom) from which the conventional electrospray source was removed and replaced by an atmospheric pressure interface^50^. Spectral acquisition was performed in positive ion resolution mode with a scan time of 1 sec. Prior to SpiderMass analysis, the samples were taken out of the -20°C freezer and thawed to RT for 30 s. The spectral acquisition sequence was composed of 2 or 3 acquisitions using 1-sec irradiation periods. The ROI were selected using the double IHC against p53 and Ki67.
