# Supplementary material for: Fallopian tube lesions as potential precursors of early ovarian cancer: a comprehensive proteomic analysis
Source: Cell Death Dis. 2023 Sep 30;14(9):644. doi: 10.1038/s41419-023-06165-5 (PMC10541450; doi:10.1038/s41419-023-06165-5)
Supplement: Supplementary file 8 — supp Data 6 [file 41419_2023_6165_MOESM8_ESM.pptx]

## Slide 1
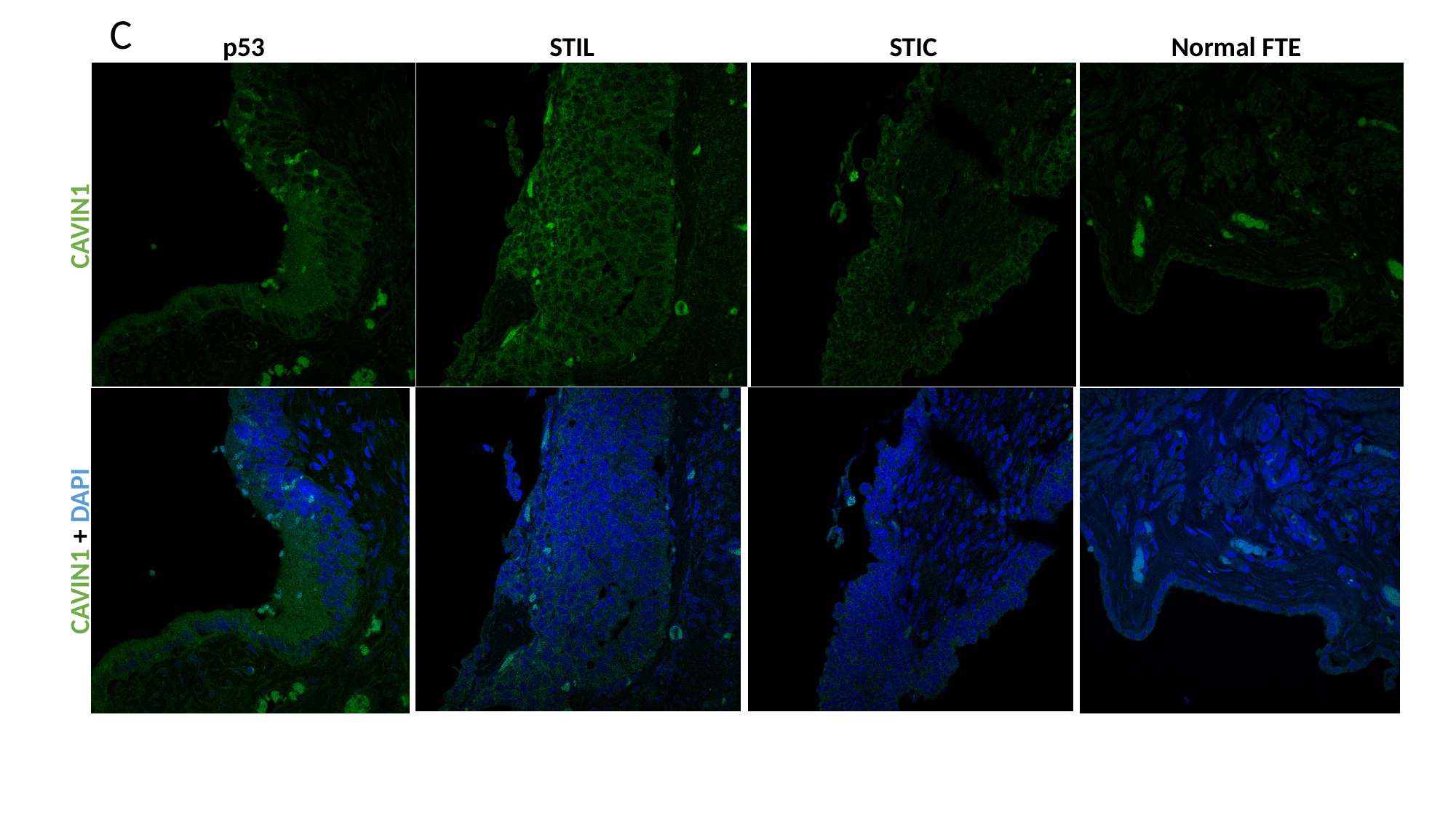

C
p53
STIL
STIC
Normal FTE
CAVIN1
CAVIN1 + DAPI

## Slide 2
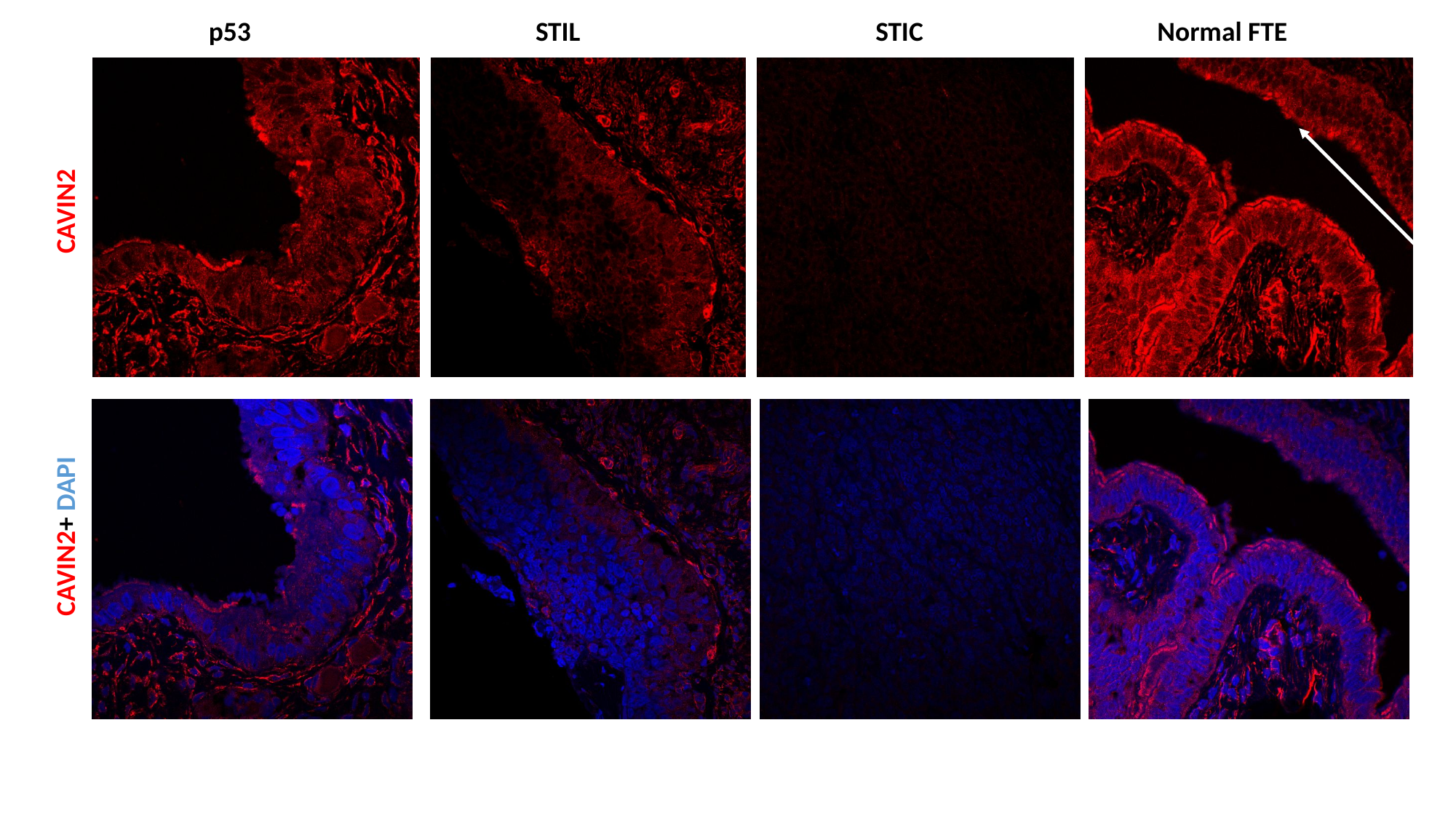

p53
STIL
STIC
Normal FTE
CAVIN2
CAVIN2+ DAPI

## Slide 3
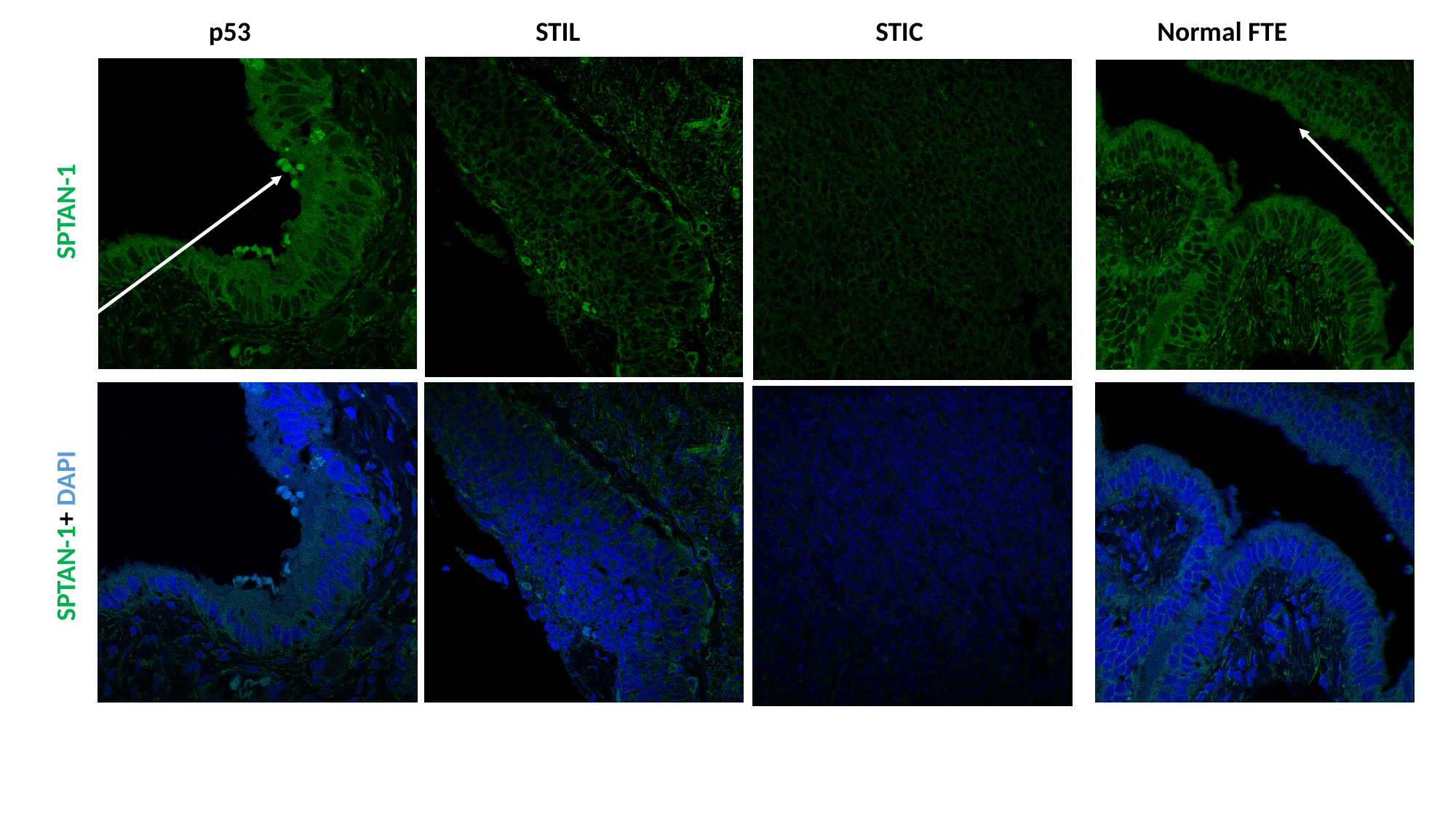

p53
STIL
STIC
Normal FTE
SPTAN-1
SPTAN-1+ DAPI

## Slide 4
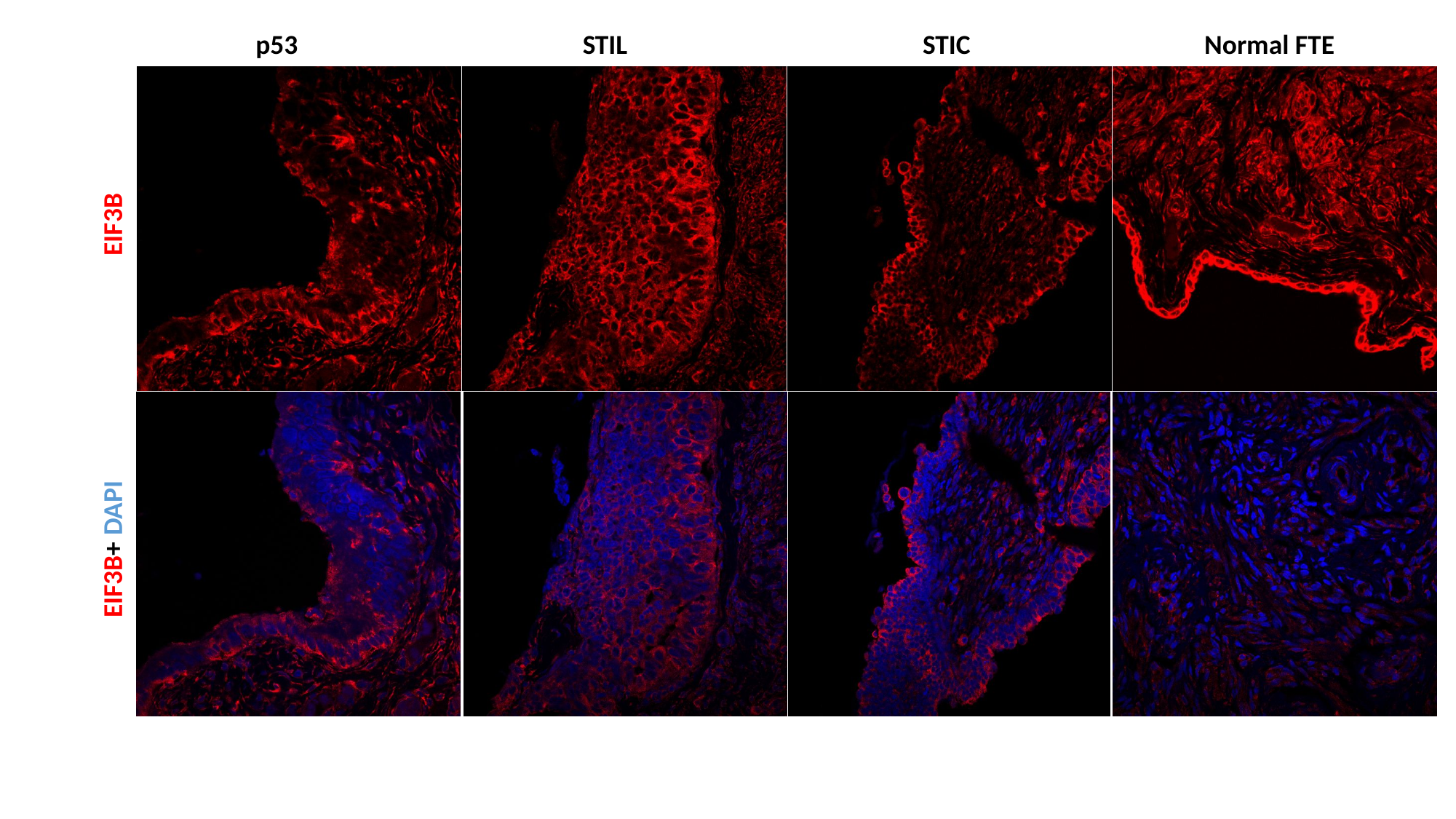

p53
STIL
STIC
Normal FTE
EIF3B
EIF3B+ DAPI
